# The Constitutive Extracellular Protein Release by Acute Myeloid Leukemia Cells—A Proteomic Study of Patient Heterogeneity and Its Modulation by Mesenchymal Stromal Cells

**DOI:** 10.3390/cancers13071509

**Published:** 2021-03-25

**Authors:** Elise Aasebø, Annette K. Brenner, Even Birkeland, Tor Henrik Anderson Tvedt, Frode Selheim, Frode S. Berven, Øystein Bruserud

**Affiliations:** 1 Department of Clinical Science, University of Bergen, 5021 Bergen, Norway; elise.aasebo@uib.no (E.A.); annette.brenner@uib.no (A.K.B.); 2 The Proteomics Facility of the University of Bergen (PROBE), University of Bergen, 5009 Bergen, Norway; even.birkeland@uib.no (E.B.); frode.selheim@uib.no (F.S.); frode.berven@uib.no (F.S.B.); 3 Department of Medicine, Haukeland University Hospital, 5021 Bergen, Norway; tor.henrik.anderson.tvedt@helse-bergen.no

**Keywords:** acute myeloid leukemia, mesenchymal stem cells, protein, proteomics, extracellular protein release, conditioned medium, patient heterogeneity

## Abstract

**Simple Summary:**

The formation of normal blood cells in the bone marrow is supported by a network of non-hematopoietic cells including connective tissue cells, blood vessel cells and bone-forming cells. These cell types support and regulate the growth of acute myeloid leukemia (AML) cells and communicate with leukemic cells through the release of proteins to their common extracellular microenvironment. One of the AML-supporting normal cell types is a subset of connective tissue cells called mesenchymal stem cells. In the present study, we observed that AML cells release a wide range of diverse proteins into their microenvironment, but patients differ both with regard to the number and amount of released proteins. Inhibition of this bidirectional communication through protein release between AML cells and leukemia-supporting normal cells may become a new strategy for cancer treatment.

**Abstract:**

Extracellular protein release is important both for the formation of extracellular matrix and for communication between cells. We investigated the extracellular protein release by in vitro cultured normal mesenchymal stem cells (MSCs) and by primary human acute myeloid leukemia (AML) cells derived from 40 consecutive patients. We observed quantifiable levels of 3082 proteins in our study; for the MSCs, we detected 1446 proteins, whereas the number of released proteins for the AML cells showed wide variation between patients (average number 1699, range 557–2380). The proteins were derived from various cellular compartments (e.g., cell membrane, nucleus, and cytoplasms), several organelles (e.g., cytoskeleton, endoplasmatic reticulum, Golgi apparatus, and mitochondria) and had various functions (e.g., extracellular matrix and exosomal proteins, cytokines, soluble adhesion molecules, protein synthesis, post-transcriptional modulation, RNA binding, and ribonuclear proteins). Thus, AML patients were very heterogeneous both regarding the number of proteins and the nature of their extracellularly released proteins. The protein release profiles of MSCs and primary AML cells show a considerable overlap, but a minority of the proteins are released only or mainly by the MSC, including several extracellular matrix molecules. Taken together, our observations suggest that the protein profile of the extracellular bone marrow microenvironment differs between AML patients, these differences are mainly caused by the protein release by the leukemic cells but this leukemia-associated heterogeneity of the overall extracellular protein profile is modulated by the constitutive protein release by normal MSCs.

## 1. Introduction

Acute myeloid leukemia (AML) is an aggressive malignancy arising from hematopoietic stem cells, and the median age at the time of first diagnosis is 65–70 years [1]. Acute promyelocytic leukemia (APL) is an AML subtype characterized by specific genetic abnormalities, different treatment and better prognosis than non-APL AML [2]. In our present study, we investigated patients with non-APL variants of the disease, and the term AML in this article therefore refers to the non-APL variants of AML [3].

AML is an aggressive malignancy; the only possibility for cure is intensive chemotherapy, possibly including allogeneic stem cell transplantation. However, patients with poor-risk genetic features have a very high risk of chemoresistant disease or relapse [1]. Many elderly and unfit patients can only receive supportive care and have a median survival of 1–3 months after diagnosis [1]. Furthermore, most elderly patients cannot receive the intensive treatment due to an unacceptable risk of severe complications and treatment-related mortality [1]. Thus, there is a need for more effective and less toxic treatment in AML that can be used in combination with the conventional intensive therapy for younger patients and/or as a less toxic treatment in elderly/unfit patients.

The proliferation and survival of primary human AML cells is supported by neighboring non-leukemic stromal cells in the bone marrow microenvironment, including the mesenchymal stem/stromal cells (MSCs) that are a part of the normal bone marrow stem cell niches [4,5,6,7,8,9,10,11]. Even though the AML cell population has a hierarchical organization and the minority of AML stem cells are regarded as essential for development of chemoresistant leukemia relapse [6], the AML-supporting extracellular bone marrow microenvironment is influenced by the total AML cell population and not only the very small minority of leukemic stem cells [7]. This is further supported by the observations from a previous study describing an association between the constitutive cytokine release profile and AML-free survival (i.e., reflecting a decreased risk of chemoresistant relapse) [8]. The constitutive extracellular protein release is probably important for the bidirectional crosstalk that can influence the functional characteristics of both the leukemic and stromal cells [9,10,11]. Our hypothesis was that AML patients are heterogeneous also with regard to the constitutive protein release by their leukemic cells, but the effect of this heterogeneity on the common extracellular microenvironment of AML and stromal cells is modulated by the constitutive release by stromal cells. In this context, we have characterized the overall extracellular protein release profiles for AML cells derived from 40 consecutive patients. We describe the variation of the in vitro constitutive extracellular protein release between patients and how the extracellular MSC protein release is modulated by primary human AML cells.

## 2. Materials and Methods

### 2.1. AML Patients and Preparation of Enriched AML Cells

Primary human AML cells were derived from the peripheral blood of 40 patients after written informed consent and in accordance with the Declaration of Helsinki (see Table 1 and Table S1). The Regional Ethics Committee approved both the collection of biological material in the biobank (REK Vest 2015/1759) and the use of the cells in the present study (REK Vest 2017/305). All AML patients had a relatively high peripheral blood level and percentage among circulating leukocytes of AML cells (AML cell concentration >15 × 10^9^/L, >80% of circulating leukocytes being AML cells), and highly enriched AML cell population (>95%) could therefore be prepared by density gradient separation alone (Lymphoprep, Axis-Shield, Norway). The cells were stored cryopreserved in liquid nitrogen until used. Only 19 patients received intensive antileukemic treatment; 4 patients died from treatment-related toxicity and 11 of the 15 patients who completed the planned intensive antileukemic treatment later died from chemoresistant relapse.

Cryopreserved human MSCs from bone marrow (> 500,000 cells) of a healthy 73-year-old female donor (C-12974; lot number 427Z010.1) were purchased from PromoCell Gmbh (Heidelberg, Germany). These cells had been cryopreserved in passage two and the MSC phenotype was verified by flow cytometric analysis and cell morphology; the proliferation potential, adherence rate and viability were also documented and the cells had the capacity to differentiate into the various mesenchymal lineages. The cells were shipped and stored in liquid nitrogen until use. The MSCs were adherent, showed a normal morphology judged by light microscopy and a typical protein release profile when cultured in serum-free medium (see Section 2.2) [12].

### 2.2. Cell Culture

Primary AML cells were cultured at a concentration of 10 × 10^6^ cells/mL (10 mL medium per flask) in T25 flasks (Falcon; Glendale, AZ); the culture medium was serum-free IMDM without phenol red (Ref. 21056023, ThermoFisher Scientific; Waltham, MA, USA). The supernatants (referred to as AML-conditioned media; AML-CM) were collected after 48 h and stored in aliquots at −80 °C, as described previously [12].

**Figure 1:**
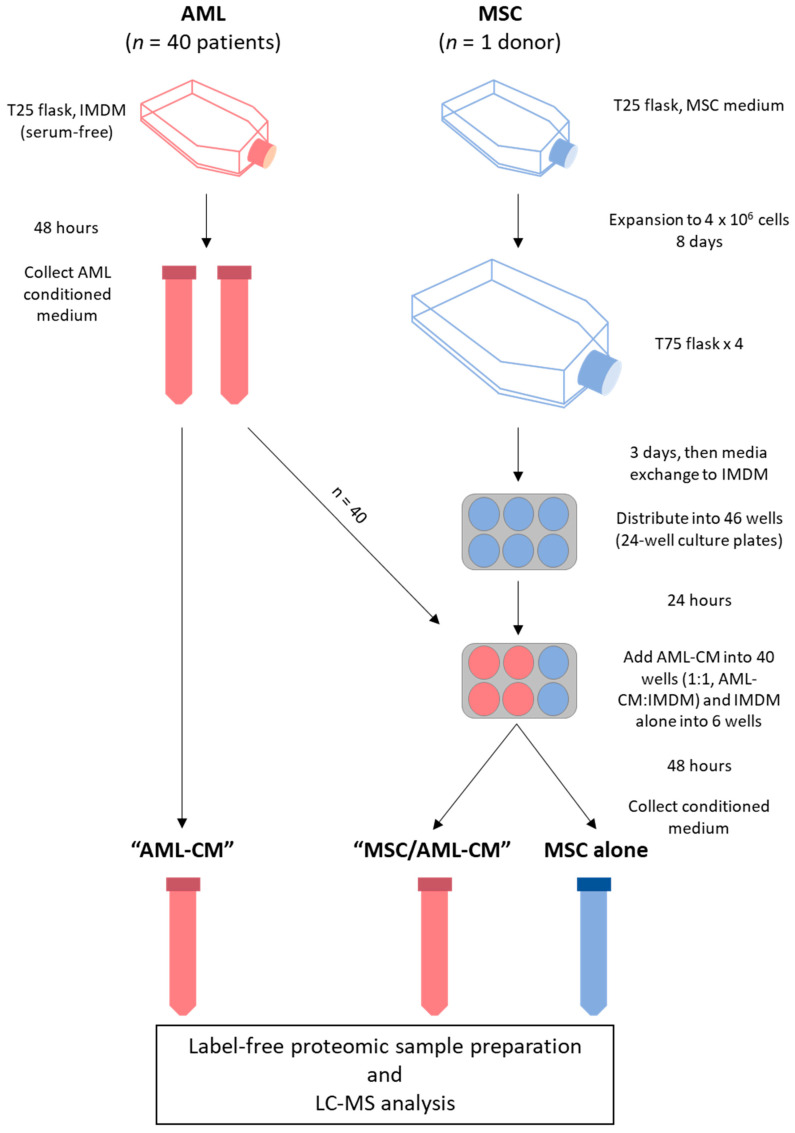
Experimental workflow. Conditioned media (CM) samples from 40 AML patient cell cultures were collected in two aliquots, of which one was analyzed alone (i.e., AML-CM) and one was added to MSC cultures derived from one donor in ratio 1:1 (i.e., MSC/AML-CM). Medium without AML-CM was added to six MSC cultures (i.e., MSCs alone). One aliquot of the MSC donor cells was also cultured under the same conditions as the AML cell cultures (not included in the figure). See Section 2.2 for details.

MSCs were thawed according to the manufacturer’s instructions and 5 × 10^5^ cells were expanded to 4 × 10^6^ cells in Mesenchymal Stem Cell Growth Medium (Promocell Gmbh) before the cells were distributed into four T75 flasks (Falcon) after eight days of culture. The cells were cultured for three additional days before the medium was changed to IMDM, thereafter transferred to 24-well culture plates (Falcon) (3 × 10^4^ cells/well) and cultured in Mesenchymal Stem Cell Growth Medium for one day before the medium was again changed to serum-free IMDM. AML-CM (also prepared in serum-free IMDM) were added at a ratio of 1:1 (supernatant:IMDM). MSCs were cultured in the presence of AML-CM for 48 h before supernatants were collected (referred to as MSC/AML-CM). Six replicates of MSCs were also cultured without AML-CM, and one aliquot of the MSC donor was cultured in a T25 flask under the same conditions as the AML cells. The supernatants were stored in aliquots at −80 °C until analyzed. An overview of the experimental workflow is summarized in Figure 1.

### 2.3. Proteomics Sample Preparation and Liquid Chromatography (LC)–Tandem Mass Spectrometry (MS/MS) Analysis

Proteomic sample preparation and liquid chromatography (LC)–tandem mass spectrometry (MS/MS) analysis has been described previously [12].

### 2.4. Statistical and Bioinformatical Analyses

Analysis of the LC–MS/MS raw files in MaxQuant (version 1.6.1.0; Max Planc Institute for Biochemistry, Martinsread, Germany) [13,14] and further data processing in Perseus (version 1.6.1.1; Max Planck Institute for Biochemistry) [15] has been described previously [12]. Funrich version 3.1.3 [16] and a GO tool [17] (selecting the filter hierarchy option for the results) was used for GO analyses, and GO terms with FDR < 0.05 were considered as significantly enriched. Graphpad Prism (version 8, San Diego, CA, USA) was used to generate correlation and bar plots. Welch’s *t*-test was used to find proteins with significantly different abundance of proteins in the groups, except for the corresponding AML-CM and MSC/AML-CM pairs where a paired *t*-test was used. Z-statistics [18] was used in addition to Welch’s *t*-test to find significantly different fold changes between the main patient clusters constructed by hierarchical clustering analysis of the AML-CM dataset (Section 3.3). Furthermore, we required a four-fold reduction or two-fold increase in protein abundance when comparing MSC/AML-CM to AML-CM because the MSC/AML-CM samples were cultured in 50% AML-CM. Protein interaction network analysis was performed with the String database (version 11.0) [19] and Cytoscape (version 3.3.0; National Institute of General Medical Sciences, Bethesda, MD, USA) [20] as previously described [12], except that MCODE (version 2.0) was used to classify densely connected protein networks of high cohesiveness [21].

## 3. Results

### 3.1. Constitutive Extracellular Protein Release by AML; Characterization of Patient Heterogeneity and the Overlap with MSC Release

**Figure 2:**
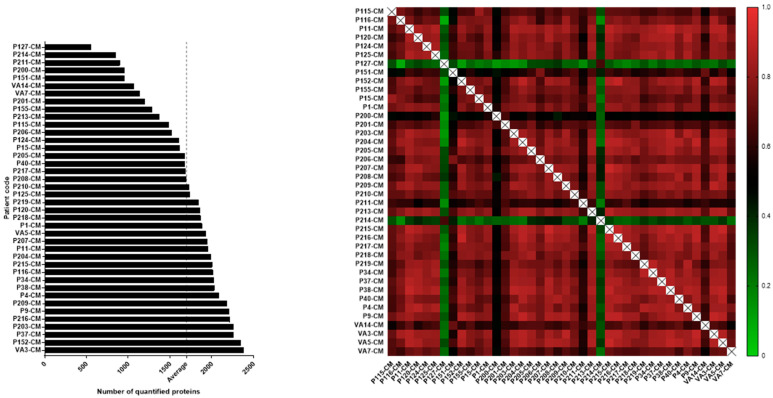
The heterogeneity in constitutive protein release by primary human AML cells; a comparison of leukemic cells derived from 40 patients. The cells were cultured for 48 h in serum-free medium before supernatants were harvested and the proteomic analyses performed. (Left figure) The figure presents the number of detectable proteins for each of the 40 patients. The number of quantified proteins varied from 557 to 2380 proteins (1699 proteins in average, vertical dotted line) between individual patients. (Right figure) The heatmap of the Pearson Correlation R values illustrates how well the protein expression correlates between the 40 AML patients, i.e., the patients showed a large degree of overlap with regard to the abundance of proteins released. This analysis is based on the 1770 proteins detected for at least 20 of the 40 AML-CM samples.

Primary AML cells from 40 patients (Table S1, Figure 1) were cultured in serum-free medium for 48 h before the supernatants were collected; these supernatants will also be referred to as conditioned medium (AML-CM). A total of 3026 proteins were quantified for the 40 patients (Supplementary File 2), but the number of quantified proteins per patient varied from 557 to 2380 proteins (Figure 2, left). The abundance of released proteins was significantly correlated between patients except for the two outlier patients P114 and P127 (Figure 2, right).

Among the 3026 different proteins released by primary human AML cells, 435 proteins were quantified in AML-CM for at least 38 patients (161 expressed in all 40 patients), whereas 415 proteins were quantified in AML-CM for five or fewer patients (98 proteins only in one patient each). Only 1770 proteins were quantified for at least 50% of the AML patients. These observations clearly illustrate that there is a considerable variation between patients regarding the number and identity of proteins released during in vitro culture.

**Figure 3:**
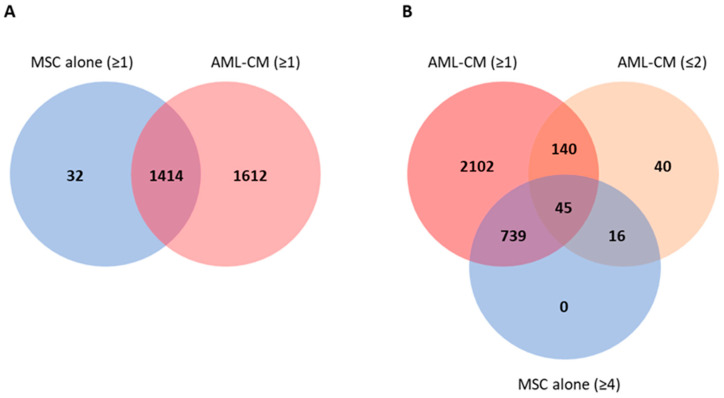
Venn diagrams of proteins quantified in MSC and AML-conditioned media samples. (**A**) Number of proteins quantified in at least one AML/MSC sample. (**B**) Number of proteins quantified in one or more AML-CM sample compared to the number of proteins quantified at least four MSC replicates (cultured alone) and in two or fewer AML-CM samples. In total, 61 proteins (45 + 16) were more often released by the MSCs as they were found in only two or fewer AML-CM samples.

We also compared proteins released by the 40 AML cell populations with the proteins released by the 7 MSC replicates derived from our healthy donor. We quantified 1446 proteins released by the MSCs, of which 800 proteins were observed for at least four of the replicate samples; these proteins are thus released at relatively high concentrations so that they reach detectable levels in most replicates despite the minor variations between these seven independent experiments/replicates. The MSCs cultured alone showed a large overlap with the AML-CM as only 32 of the 1446 proteins were not quantified for any AML patient (Figure 3A). A total of 61 MSC proteins were quantified for two or fewer of the AML patients but at least four MSC cultures (Figure 3B, Table S2), and 16 of these were not released by any AML cell population in our study. Thus, the MSCs released relatively few unique proteins compared with many primary AML cell populations, and there was in addition a large overlap between leukemic cells and MSCs in their extracellular protein release with only a minority of the MSC-released proteins showing undetectable levels for all or most AML patients. Furthermore, we found several interacting proteins in an interaction network analysis based on the 61 proteins predominantly released by MSCs, and the proteins were annotated to GO terms as secreted, signal and extracellular matrix (Figure S1). The 16 MSC-specific proteins as well as the proteins detected only for two or fewer AML patients were very heterogeneous and included extracellular matrix proteins, cytokines and cytokine receptors, soluble adhesion molecules and proteases (Table S2 and Figure S1).

### 3.2. Primary Human AML Cells Release of Proteins Derived from Different Cellular Compartments

GO analysis (using FunRich) of cellular compartment based on all identified proteins showed that 68% were annotated to the cytoplasm, 55% to the nucleus and 44% to exosomes (Table 2). More than 300 proteins were also annotated to the plasma membrane. Thus, the released proteins are derived from various cellular compartments or intracellular organelles. Analysis of the GO terms with regard to molecular functions also demonstrated that the released proteins are very heterogeneous (Table S3). Large subsets of proteins were classified as ribosomal/RNA binding proteins, DNA binding proteins, transcriptional regulators, cytoskeleton binding protein or having catalytic, chaperone or transporter activity. Finally, the most significant biological processes identified through GO term analyses were protein metabolism (322 proteins, corrected *p*-value 5.87 × 10^−57^), energy pathways (*p*-value 3 × 10^−18^) and cell growth and/or maintenance (*p*-value 0.00049). Taken together, these last analyses indicate that the AML cells release a wide range of non-extracellular matrix proteins derived from various intracellular compartments and reflecting different intracellular processes.

### 3.3. Subclassification of AML Patients Based on the Constitutive Protein Release Profile of Their In Vitro Cultured Leukemic Cells

**Figure 4:**
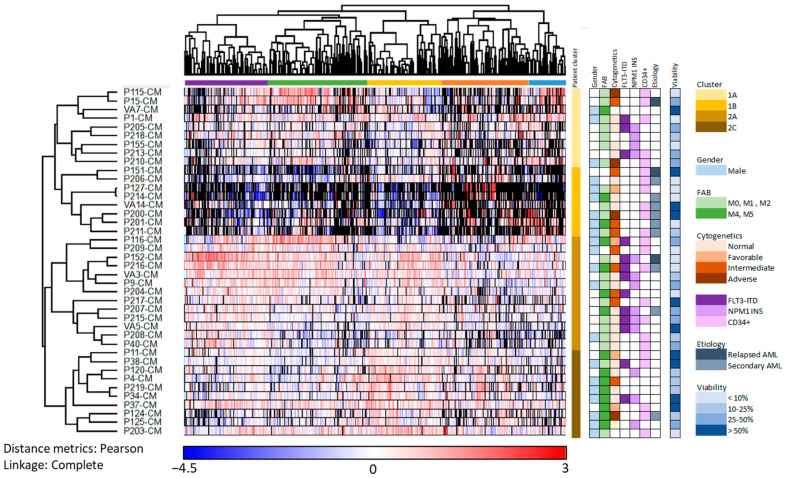
Identification of AML patient subsets based on their constitutive protein release during in vitro culture; an unsupervised hierarchical cluster analysis including 40 patients and based on 1770 proteins detected for at least 50% of the patients. The cells were cultured for 48 h in serum-free medium before supernatants were harvested and the proteomic analyses performed. The patients clustered into two main clusters (yellow/upper and brown/lower; see the column on the right side of the clustering); each of these two main clusters had two subclusters (upper yellow/dark yellow and lower brown/dark brown, respectively). As can be seen from the upper part of the figure, the proteins clustered into five main clusters each including 392 proteins (Cluster 1, purple), 460 proteins (Cluster 2, green), 337 proteins (Cluster 3, yellow), 417 proteins (Cluster 4, orange) and 164 proteins (Cluster 5, blue), respectively. Patient characteristics are indicated to the right in the figure, and blank fields indicate information not determined. Black color in the cluster analysis indicates that the protein was not detected.

In the present study, we included consecutive AML patients which were heterogeneous in terms of clinical and biological characteristics (Table S1). We performed a hierarchical clustering analysis based on the 1770 of the 3026 AML-released proteins that could be quantified for at least 50% of the patients; the influence of exceptional proteins/patients on this analysis of protein/patient subclassification was thereby reduced (Figure 4, Supplementary File 2). These released proteins formed five different clusters as indicated by the colors at the top of Figure 4 (purple, green, yellow, orange and blue clusters), and the enriched GO terms differed between each of these five protein clusters (Table 3).

The left purple cluster especially included proteins from the cytoplasm or various cytoplasmic organelles, the middle left green cluster especially nuclear proteins, the middle yellow cluster especially exosomal/vesicular/extracellular proteins, the orange middle right cluster especially extracellular matrix proteins as well as proteins involved in intracellular vesicle-mediated transport, while no GO terms were significantly enriched in right blue cluster. The clustering analysis (Figure 4) identified two main patient clusters indicated by yellow (cluster 1; upper) and brown color (cluster 2; lower) to the right in the figure. Each of the two main patient clusters included two subclusters indicated by bright yellow/yellow and brown/dark brown, respectively. The four subclusters differed significantly when analyzing the number of quantified proteins in each cluster (Kruskal–Wallis test, *p* = 0.0001); patients in the lower brown main cluster generally released higher numbers of proteins (cluster 2; 23 patients, median number 2030 proteins, range 1686–2380) compared with the patients in the upper yellow main cluster (cluster 1; 17 patients, median number 1282, range 557–1864, Wilcoxon’s test, *p* = 0.0034).

**Figure 5:**
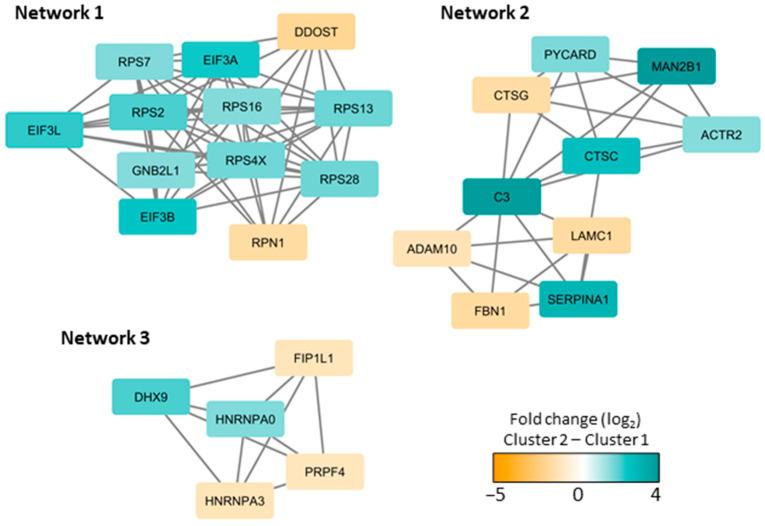
Densely connected protein interaction networks based on the proteins with significantly different protein abundances in cluster 1 (see Figure 4, upper yellow main cluster) and cluster 2 (see Figure 4, lower brown main cluster) in Figure 4. A large protein interaction network was generated in String and imported into Cytoscape to find densely connected proteins using the MCODE application (see Figure S2 for the complete network). The color coding indicates the protein fold change (log_2_ transformed) between cluster 2 and 1, where turquoise illustrates increased abundance and orange illustrates decreased abundance in the lower main cluster 2.

A statistical analysis based on the proteins in the two main clusters (Figure 4; main yellow/cluster 1 and brown/cluster 2) resulted in 144 proteins with significantly different protein abundance, using Welch’s *t*-test and Z-statistics. Several interacting proteins were identified in interaction network analyses (Figure S2), and three networks showed densely connected proteins (Figure 5). Network 1 was dominated by ribosomal proteins and included 12 proteins mainly belonging to the purple protein cluster (see Figure 4, upper part). Network 2 was enriched with proteins annotated to secretory granules and with all ten proteins belonging to the middle yellow or orange protein clusters (Figure 4). The mRNA processing proteins in network 3 did not belong to any specific protein cluster. The patients in cluster 2 (lower brown cluster in Figure 4) thus seem to have higher abundance of several ribosomal and secretory granule proteins compared to the patients in cluster 1 (upper yellow cluster).

The difference in the number of released proteins between the two yellow/upper subclusters reached only borderline significance (*p* = 0.0455), the patients in the bright yellow upper subcluster showing reduced release especially of proteins in the middle right brown protein clusters (exosomal/extracellular and nuclear proteins). In contrast, the patients in the lower yellow subcluster showed a generally reduced number of released extracellular proteins, and this patient subcluster also included a significantly increased number of patients with secondary AML (CMML or MDS; Fisher’s exact test, *p* = 0.0197). As would then be expected this cluster also included a larger fraction of patients above 70 years of age (7/8 versus 14/18; Fisher’s exact test, *p* = 0.0461); younger patients were especially seen in the lower dark brown cluster. Furthermore, the patient clusters did not differ significantly with regard to gender, differentiation (FAB classification/CD34 expression) karyotype or Flt3/NPM1 abnormalities. Finally, the patients in the lowest dark brown subcluster showed high levels of proteins in the middle yellow protein cluster that especially included exosomal/vesicular/extracellular proteins (e.g., collagen trimer and endoplasmic reticulum proteins). Thus, AML patients can be further subclassified into distinct subsets based on the number and nature of constitutively released proteins by their AML cells, and these differences correspond to differences in important functional cell characteristics.

**Figure 6:**
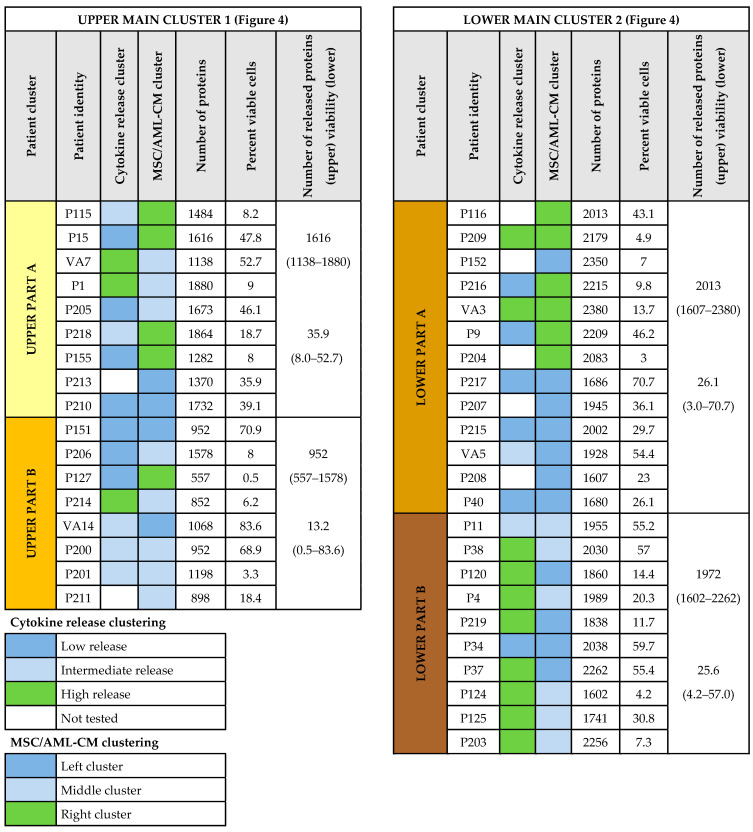
Identification of AML patient subsets by hierarchical clustering analysis of constitutive leukemic cell protein release. The AML cells were derived from 40 patients, and the cells were cultured alone for 48 h in serum-free medium before supernatants were analyzed. The number of quantified proteins varied from 557 to 2380 proteins (average 1699 proteins), and the proteomic analysis was based on the 1770 proteins detected in 50% or more of the AML-CM samples. This clustering analysis is presented in Figure 4; two main clusters each with two subclusters were identified (left and right part, respectively, of the present Figure 6), and the patients are listed from the upper part to the bottom of the present figure according to the results from this clustering analysis (patient identity columns). For 33 patients we also analyzed their release of 19 selected soluble mediators for AML cells cultured alone using antibody-based methodology (Figure S3); a hierarchical clustering analysis based on these mediator levels classified the patients into three main subsets with generally low, intermediate and high constitutive mediator release (see upper right). The column Cytokine release cluster refers to this classification, and the color codes are explained in the lower left part of the figure. Finally, we also did a hierarchical clustering analysis based on the protein release profile of all 40 patients when MSCs were cultured with AML-CM for all 40 patients (Figure S4). The subclusters/subclassification of patients based on this last analysis is summarized in the column referred to as MSC/AML-CM cluster (color code explanation, see lower left). The right part of the figure presents the number of quantified proteins and the number of viable AML cells after 48 h of in vitro culture for each patient sample. The median and range of quantified proteins/viability for each of the four patient subsets are presented to the right in each part of the figure.

Previous studies have shown that AML patients differ with regard to the proliferative capacity and the degree of spontaneous apoptosis during in vitro culture of their leukemic cells [22,23]. However, the proliferative capacity after seven days of in vitro culture and the percentage of viable cells after two days of culture did not differ significantly between the two main patient clusters or between the subclusters (see Figure 4 and Figure 6) identified by the cluster analysis presented in Figure 4. Thus, even though protein release by necrotic/apoptotic cells during in vitro culture may contribute to the extracellular protein release of our cultured AML cells, such differences seem to be relatively small and cannot explain the patient subset classification identified in Figure 4.

AML patients differ in the capacity of their leukemic cells to constitutively release cytokines/chemokines and proteases/protease inhibitors during in vitro culture and based on these differences patients can be classified as showing generally high, intermediate/variable and low constitutive release of these mediators [8,24,25,26,27]. Soluble mediator release data were available for 33 (unselective/consecutive) of our 40 AML patients (i.e., antibody-mediated estimation of soluble mediator levels in supernatants), and these results confirmed that patients could be classified into three main subsets based on their capacity of constitutive cytokine release (Figure S3). High constitutive cytokine release was observed especially for patients in the lowest dark brown patient subcluster characterized by generally high constitutive protein release (8 out of 10 patients); this is significantly different from the other patients (5 out of 17, Fisher’s exact test, *p* = 0.0183). Thus, the release of a high number of extracellular proteins (i.e., a characteristic of the lower brown main cluster, see above) is associated with a high capacity of cytokine/chemokines and proteases/protease inhibitor release.

### 3.4. The AML-Associated Heterogeneity of the Extracellular Protein Profile Is Largely Maintained also in the Presence of MSCs

Most of the quantified proteins released by MSCs were also released by primary AML cells, but as described above, the number of AML cell populations with detectable levels of each individual protein showed wide variation. MSC cultures were therefore prepared in medium alone and in medium supplemented with AML-CM (referred to as MSC/AML-CM) (Figure 1). All 40 leukemia patients were included in these experiments, and we first did a correlation analysis including all detectable proteins and all 40 patients to investigate whether the presence of protein-releasing MSCs reduced the patient heterogeneity. We observed a strong correlation between the number of patients with detectable levels of individual proteins in the AML-CM and in the supernatants from MSC cultures supplemented with the corresponding AML-CM (Spearman Rank Correlation test, r = 0.8019, *p* < 0.00005). Thus, for the large majority of released proteins the fraction of patients with detectable levels is comparable when AML cells are cultured alone (i.e., AML-CM) and when MSCs are cultured with the same AML-conditioned medium. Finally, we also performed similar correlation analyses based on protein subsets that are important for cellular communication or adhesion, and similar strong associations were also observed when only including defined protein subsets, i.e., 80 extracellular matrix molecules (Spearman’s rho, 0.667), 67 soluble extracellular mediators (cytokines/chemokines/growth factors (Spearman’s rho, 0.803) and 300 proteins included in the GO term Protease (Spearman’s rho, 0.785).

### 3.5. Reduction in Patient Heterogeneity by MSCs; a Small Subset of Proteins Show Heterogeneous AML Cell Release but Are Released at High Levels by MSCs

Even though patient heterogeneity seems to be largely maintained even in the presence of MSCs (see Section 3.4), we identified a minority of 60 deviating proteins (Table 4); these proteins were released at quantifiable levels only for 10 or fewer of the 40 patients when AML cells were cultured alone (i.e., in AML-CM) but showed quantifiable levels for at least 30 patients when MSCs were cultured with AML-CM. All these 60 proteins were released at high levels when MSCs were cultured alone, and they included 24 extracellular matrix proteins (5 collagens), 10 cell surface/adhesion molecules and 8 enzymes (see detailed description in Table S4).

Previous studies have identified several stroma-derived molecules that are important for the maintenance and function of normal hematopoietic stem cells in the bone marrow stem cell niches [28,29,30,31,32]. It can be seen from Table 4 that several of these stem cell regulating proteins (e.g., several extracellular matrix and adhesion molecules) were included among the 60 proteins showing undetectable levels for most patients when AML cells were cultured alone but detectable levels for most MSC cultures supplemented with the corresponding AML-CM (marked in Table 4; five extracellular matrix molecules, three enzymes important for post-transcriptional modulation of matrix molecules, three soluble cadherins). A total of 795 proteins showed detectable levels for 10 or fewer of the 40 patients when the AML cells were cultured alone; these 795 proteins reflect a heterogeneity between patients with regard to their constitutive protein release and this heterogeneity is reduced/eliminated only for 60 of these proteins when MSCs are present. Thus, the presence of MSCs has a limited effect on this heterogeneity of the overall extracellular protein release by primary human AML cells.

We performed an additional GO term analysis (using a GO tool) of these 60 proteins found in 10 or fewer AML-CM samples but in at least 30 MSC/AML-CM samples. The results of the analyses of cellular compartment and molecular functions are presented in Table 5. Several highly significant GO terms were identified in all analyses. These terms mainly reflect that the 60 proteins have important extracellular function and being either extracellular matrix molecules or modulators of the extracellular matrix. Several proteins are also important for cell–cell or cell–matrix adhesion, are localized to the cell surface membrane or are important for the binding of soluble mediators. Finally, the same biological functions as in the analysis of cellular compartments/molecular functions were also reflected identified in GO term analyses of biological processes and additional analyses based on the KEGG and Uniprot databases. Taken together, this dominance of certain protein subsets among the 60 proteins clearly illustrates that they are not identified by coincidence, and the increased levels of these proteins are not caused by a random process.

Taken together, these analyses support the conclusion from Section 3.4. and suggest that the presence of MSCs has a limited effect on the AML-associated heterogeneity of the extracellular protein profile of the common MSC/AML cell microenvironment, i.e., only 60 of 795 proteins released for 10 or fewer patients are detected in the supernatant samples for most patients in the presence of MSCs (see Table S4).

### 3.6. The Effect of MSCs on the AML-Associated Heterogeneity of of Their Common Extracellular Protein Profile; Relatively Few of the Quantified Proteins Are Significantly Altered by the Presence of MSCs

**Figure 7:**
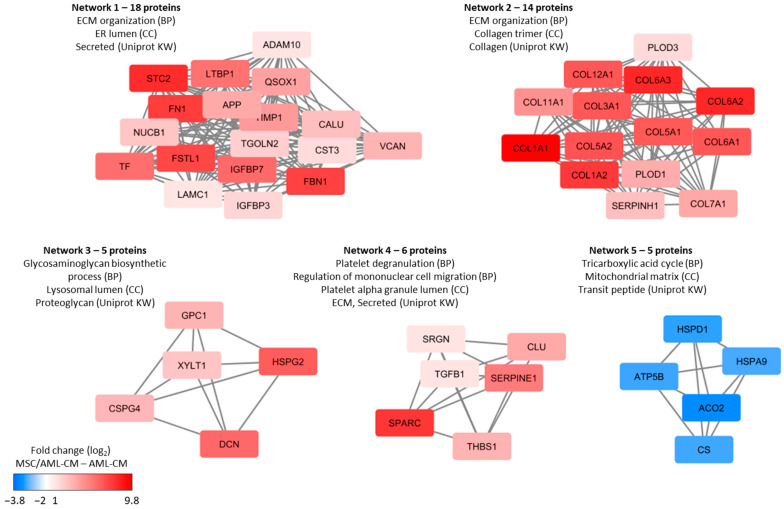
Densely connected protein networks based on proteins which were two-fold increased or four-fold decreased in the MSC/AML-CM culture supernatants compared to AML-CM (i.e., corresponding AML cells cultured alone). Proteins with significantly altered protein abundance were imported to String for protein interaction network analysis. The protein networks were further imported to Cytoscape where the MCODE application was used to find closely connected protein networks containing at least five proteins. The GO annotations were identified by the String database. Red color indicates increased abundance in MSC/AML-CM and blue color indicates decreased abundance in MSC/AML-CM relative to AML-CM. BP, biological processes; CC, cellular compartment; Uniprot KW, Uniprot Keywords; ECM, extracellular matrix; ER, endoplasmic reticulum.

To further investigate the additional contribution of MSCs to the in vitro protein release profile when these cells were influenced by the heterogeneous AML cells (i.e., incubated with AML-CM), we compared the levels of individual proteins in supernatants from MSC/AML-CM cultures with the corresponding AML-CM. Only the 2304 proteins with quantitative levels for at least four pairs of AML-CM and MSC/AML-CM were included in this analysis. As the AML-CM was added to the MSCs at a 1:1 ratio, we assumed a lower abundance of the AML-CM-derived proteins in the MSC/AML-CM samples compared to AML-CM alone. For this reason we defined a significant effect by a fold change criterion (i.e., of two-fold increase or four-fold decrease in MSC/AML-CM relative to AML-CM) together with a statistical criterion (i.e., *p*-value < 0.05, paired *t*-test). When analyzing the overall results, we observed significantly increased abundance corresponding to more than a two-fold increase for 146 proteins in the MSC/AML-CM cultures whereas only 26 proteins showed decreased abundance corresponding to at least a four-fold decrease (Table S5). Thus, the presence of MSCs causes a significant quantitative alteration in the extracellular levels only for a minority of the quantified proteins. Protein interaction network analysis of these differently released proteins showed that the proteins with increased levels in MSC/AML-CM cultures relative to the corresponding AML-CM samples were involved especially in extracellular matrix (ECM) organization but also related processes including glycosamin biosynthesis, extracellular protein release (i.e., platelet degranulation) and regulation of metabolism (Figure 7).

MSCs cultured in medium without AML-CM showed detectable release for 140 of the 146 proteins that had significantly increased abundance in MSC/AML-CM culture supernatants; thus, only a small minority of these proteins (CD82, ADAM17, SCP2, S100B, CPNE3 and LRMP) was not detected in the MSC supernatants.

To conclude, these analyses described in this section further support the main conclusions from Section 3.4 and Section 3.5; i.e., the presence of MSCs has a limited effect of the AML-associated heterogeneity of the extracellular protein profile in the common microenvironment. In Section 3.4 and Section 3.5, we described qualitative differences caused by MSCs, whereas we analyzed MSC-induced differences for quantifiable proteins in the present section. Additionally, for these proteins, we observed MSC effects, especially on extracellular levels of matrix protein, but in addition the levels of mitochondrial matrix proteins involved in the tricarboxylic acid cycle were altered (Figure 7 and Table S5).

### 3.7. Identification of AML Patient Subsets by a Clustering Analysis Based on the Protein Profile of Supernatants Derived from MSC Cultures Supplemented with AML-Conditioned Media

We performed a hierarchical quantitative protein clustering analysis based on the 40 supernatants derived from cultures of MSCs in the presence of AML-CM. The clustering analysis is presented in Figure S4 and a summary of these results is included in Figure 6. Based on this analysis three patient clusters/subsets were identified (Figure S4), and these clusters/subsets differed from the clusters identified in the analysis based on the AML-CM alone (Figure 4). However, the dark brown/lower patient subcluster from the AML constitutive release analysis (see Figure 4) included only patients from the left and middle patient subclusters but no patients from the right of the MSC/AML-CM analysis (Figure S4). This association between patients included in the lower dark brown (Figure 4) and right cluster (Figure S4) reached statistical significance (Fisher exact test, *p* = 0.0038), an observation further illustrating that patient heterogeneity detected in the proteomic analysis of the constitutive extracellular AML cell release (Figure 4) is at least partly maintained and thereby influences the extracellular proteomic profiles when MSCs are cultured in the presence of AML-CM. Our previous conclusion (Section 3.4, Section 3.5 and Section 3.6) is therefore supported by this new clustering analysis; i.e., the presence of MSCs does not cancel out the AML-associated heterogeneity with regard to the extracellular protein profile of their common microenvironment (Figure 4). Finally, the patient clusters/subsets identified in this new clustering analysis (Figure S4) did not differ significantly with regard to age, gender, differentiation (FAB classification/CD34 expression), karyotype, Flt3/NPM1 abnormalities or etiology (secondar versus de novo) (data not shown).

### 3.8. Both MSCs and AML Cells Show Extracellular Release of a Wide Range of Exosomal Proteins

We compared the proteins in our entire dataset to proteins annotated to the GO term Exosome, and found 1043 overlapping proteins (Supplementary File 2); 340 of these proteins were not expressed by the MSCs, whereas constitutive release of 729 of these proteins were detected in at least 20 supernatants from AML cells cultured alone (i.e., AML-CM) and 127 proteins showed detectable levels in all 40 patient samples. Table S6 shows all exosomal proteins detected in AML-CM, and we have marked with yellow all proteins that are included in the top 100 ranked exosomal proteins (http://exocarta.org/exosome_markers_new, accessed on 15 November 2020). All AML cells expressed 41 of these top-ranked proteins, an observation suggesting that exosomal release is an important mechanism for extracellular protein release by primary human AML cells.

When comparing the overall results for these 1043 exosomal proteins, significantly increased levels were detected only for 75 of them when MSC/AML-CM were compared with the corresponding AML-CM. However, the levels in the MSC/AML-CM cultures were significantly 2-fold increased for 187 proteins when compared with MSCs cultured alone, whereas 2-fold decreased levels were detected only for 101 proteins (only 14 proteins showing four-fold reduction). Thus, exosomal release seem to be maintained as an important mechanism of extracellular protein release also in MSC/AML-CM cultures.

### 3.9. Culture of MSCs in the Presence of AML-CM; the Supernatant Levels of MSC-Specific Proteins Are Decreased in the Presence of AML-CM

The protein release profiles of the MSCs were highly reproducible in repeated independent experiments (Figure S5), and we identified 61 MSC-specific proteins (i.e., proteins detected in AML-CM for ≤2 patients and in MSC cultured alone for ≥4 samples, Table S2). However, when we investigated supernatants from MSC cultures supplemented with AML-CM (i.e., MSC/AML-CM) from all 40 patients only 9 of these 61 proteins were detected for ≥30 patients, 28 proteins for 20–29 patients, 10 proteins for 10–19 patients, 8 proteins for 5–9 patients and 5 proteins for fewer than five (7 proteins) or none (1 proteins) of the patients. Thus, even though all these proteins were released at detectable levels only or mainly by MSCs, for several of them the detectable levels could only be quantified for a minority of the patients when AML-CM was present during culture. Thus, AML patients are heterogeneous also with regard to their effects on the constitutive release of MSC-specific proteins.

We also performed statistical comparisons of the abundance of MSC-specific proteins for MSC/AML-CM versus MSC cultures prepared in medium alone. None of these proteins were significantly increased when AML-CM was present, whereas 45 proteins (including 3 isoforms of POSTN) were significantly decreased compared with MSC medium controls (Table S7). Thus, the AML effect on MSC-associated protein release differs between patients (see above), but the effect of this intercellular crosstalk also differs between proteins with decreased release for a subset of proteins including several extracellular matrix molecules.

Previous studies of ECM expression at the mRNA level have described a 15-ECM gene expression signature that is associated with survival for patients receiving intensive antileukemic treatment [33,34,35]. Ten of the proteins encoded by these 15 genes could be detected in our present proteomic studies (Table S8), and five of them were not released at detectable levels by MSCs. The release of these ten detectable proteins varied between AML cells/patients; Col18A1 was only released at detectable levels for five patients whereas myeloblastin was detected for 31 patients. Furthermore, we investigated CD44 release in addition to the ECM signature proteins because it can bind to a wide range of extracellular matrix molecules [36,37,38,39,40,41,42,43], it seems to be an important normal stem cell regulator in the endothelial niche [43] and it is involved in interactions between AML cells and AML-supporting endothelial cells [42]. It is also associated with a chemoresistant AML cell phenotype [44,45] although it is not regarded as an established prognostic factor in routine clinical practice [1]. The soluble form of CD44 was released both by MSCs and by AML cells derived from 39 out of 40 patients. Only three of these 11 proteins showed significantly altered levels in MSC/AML-CM cultures compared with AML-CM and only one (ADAM17) when considering proteins with two-fold increase or four-fold decrease (Table S8). Thus, MSCs seem to have only a limited effect on this AML-associated ECM signature that possibly has a prognostic impact.

## 4. Discussion

Extracellular protein release is important for the formation of extracellular matrix and for communication between neighboring cells both in normal and leukemic hematopoiesis. In the present study, we used a proteomic strategy to characterize and compare the constitutive protein release by AML cells derived from 40 consecutive patients, and we investigated how this heterogeneity is modified by normal bone marrow MSCs. AML cells generally released a higher number of quantifiable proteins than MSC, but this release varied considerably between patients both regarding the number and nature of the quantified proteins.

In this study we used highly enriched cell populations of MSCs derived from a healthy individual. Furthermore, the enriched AML cells were derived from a consecutive group of patients with a high percentage and/or absolute number of leukemic cells among circulating leukocytes; highly enriched AML cell populations could therefore be prepared by density gradient separation alone [9,46,47]. Flow cytometric analyses confirmed that at least 90% of these gradient-separated cells were AML cells. Therefore, our results may only be representative for the subset of AML patients with circulating leukemic cells but not for patients without circulating blast cells. However, since the level of circulating AML cells has a relatively weak prognostic impact in patients receiving intensive and potentially curative treatment [48,49,50,51,52], our present results may be representative with regard to clinical chemosensitivity also for other patients. Finally, due to this consecutive selection, a large subset of our patients were elderly and/or unfit, and therefore only 19 patients received intensive and potentially curative therapy [1]. Four of these 19 patients died from toxicity, i.e., before the planned intensive treatment was completed. Due to the relatively low number of patients that completed the intensive therapy, survival analyses could not be included in this study.

Many studies suggest that AML relapse is derived from residual leukemic stem cells (for a detailed discussion and additional references see [5,53]), and the risk of relapse will therefore depend on the chemosensitivity of the minority of AML stem cells. However, the genetic abnormalities and the biological characteristics associated with these abnormalities (including chemosensitivity [1]) also seem to be reflected not only in the leukemic stem cells but also in the majority of more mature cells in the hierarchically organized AML cell population. Several studies have therefore demonstrated that the biological characteristics of the total AML cell population are associated with relapse risk and survival, including both mRNA gene expression profiles, noncoding RNA profiles, proteomic and phosphoproteomic profile as well as epigenetic and metabolic regulation [54,55,56,57,58,59,60,61]. For these reasons we regard investigation of the overall AML cell populations in our present study to be relevant.

The proteins in the AML culture supernatants (i.e., the AML-conditioned media) are released through various mechanisms. First, proteins can be released by exocytosis or the release of exosomes and this is probably the case both for AML cells and MSCs because several exosomal proteins were detected in the conditioned media. Some of the extracellular matrix proteins are very large (e.g., collagens) and recruitment of specialized proteins for packing of these molecules may be required for vesicle formation and subsequent extracellular release [62]. Second, release caused by proteolytic cleavage of cell surface molecules is also possible. Finally, we did not see increased levels of necrotic cells in the MSC cultures, but the primary AML cells showed expected spontaneous or stress-induced in vitro apoptosis during culture [23]. However, even though we cannot exclude that protein release from apoptotic/necrotic cells contributes to the protein release profiles, in our opinion, this mechanism is probably less important because the clustering or subclassification of patients (Figure 4) did not show any significant association with the cell viability at the end of the culture period.

We performed an unsupervised hierarchical clustering analysis based on the constitutive protein release of the primary AML cells (Figure 4). This analysis was based on those proteins that could be detected for at least 50% of the patients. We identified two main clusters, and each of them could be further divided into two subclusters. Our patient group is relatively small and we could only detect an association between this patient subclassification and secondary AML, whereas no significant associations with genetic abnormalities could be detected. However, the associations between protein release patient clusters and secondary leukemia [63,64] as well as constitutive cytokine release [8] suggest that the overall protein release profile has a prognostic impact.

In a previous study, we observed that the constitutive AML cell release of cytokines and proteases/protease regulators varied considerably between patients, and we could identify three patient subsets characterized by generally high, intermediate and low constitutive release, respectively [8]. Generally high constitutive release was then associated with a favorable prognosis and increased overall survival. We could investigate the constitutive cytokine release in this experimental model for a subset of 33 consecutive patients, and a cluster analysis of these data confirmed that the patients formed three main clusters (Figure S3), as described previously [8]. Furthermore, the subclassification of patients based on the more limited constitutive cytokine/protease/protease regulators release was significantly associated with the subclassification based on the general protein release profile (Figure 4 and Figure S3). Taken together, these results suggest that generally high extracellular protein release (including high constitutive cytokine release) is a part of a functional AML cell phenotype that shows high clinical chemosensitivity and thereby a favorable prognosis. A possible hypothesis for the mechanisms behind this observation could be that these patients/AML cells are more dependent on communication with and thereby external support from extracellular matrix and/or neighboring cells for proliferation and survival, thus a dependency making them more chemosensitive.

Both MSCs and primary AML cells released a wide range of proteases or regulators of proteases. Protease activity can be important for the communication between cells through several mechanisms; the contribution of the various mechanisms has been investigated more in detail for certain proteases (e.g., the ADAM family) [25,65], whereas less is known for many others with regard to their possible roles in cellular communication. First, proteases are important for the regulation of cytokine activity; cytokines can be activated through proteolytic processing, as described for several chemokines and members of the IL1 family [65,66]. Both the present and previous studies show that chemokines as well as IL1 family members can be constitutively released by primary AML cells [8,26,67]. The effects of various proteases on IL1 family cytokines with the possibility of cleavage at many different sites clearly illustrate the complexity of protease effects on cellular communication [66]. Proteases are also involved in the activation of hepatocyte growth factor [68]. Second, certain serine proteases can trigger both cytokine expression/release and cleavage; this has been described both for IL6, TNFα and IL8/CXCL8 (all three can be secreted by AML cells), and such protease effects can be mediated by Toll-like receptors (TLRs) that also are expressed by primary AML cells [69,70]. Proteases and cytokines can also be colocalized in the same secretory vesicles [71]. Third, certain proteases or protease inhibitors can function as signaling molecules and/or activate receptor signaling [72]. Finally, several ADAM proteases can cleave a wide range of membrane molecules, e.g., cytokine receptors and adhesion molecules with biological activity, and processing of extracellular or transmembrane protein domains can lead to release of intracellular domains with subsequent signal transduction [73,74]. Thus, proteases can be important for cellular communication through several mechanisms, including the communication between leukemic cells and their stromal cells/molecules. Further studies are therefore needed to clarify which role the various proteases play in leukemogenesis.

In our present study, we compared the constitutive protein release by primary AML cells (i.e., protein abundance in AML-CM) with the protein abundance when MSCs were cultured in the presence of these conditioned media from all 40 patients. A small subset of proteins showed detectable release by the leukemic cells only for a small minority of patients, whereas detectable release was observed for most patients when investigating the MSC/AML-CM culture supernatants. Even though our analysis of the overall protein release profiles suggests that patient heterogeneity is mainly maintained in the presence of MSCs, the heterogeneity of the overall extracellular protein release is reduced by the crosstalk between MSCs and primary AML cells mediated through the extracellular release of protein mediators; the MSC release decreases the heterogeneity especially for extracellular matrix molecules but also for several proteases (i.e., protein modifiers) and soluble adhesion molecules (Table 4). Many of these extracellular proteins are important for the function of bone marrow stem cell niches (Table 4) and may also be important for the function of these niches in leukemogenesis and/or cancer metastasizing [28,29,30,31,32].

As discussed above, cells can release proteins to the extracellular space through various mechanisms, e.g., proteolysis of surface proteins and through exosomes [75,76]. In the present study, we did not analyze exosomes separately and for this reason we cannot evaluate the relative contribution of each of these two mechanisms, but the high extracellular levels of several proteases suggest that proteolytic cleavage of surface molecules contributes [65,66,67,68,69,70,71,72,73,74]. However, many of our detected proteins belong to the GO term Exosomes, an observation suggesting that exosomal release is also involved. This is further supported by the high number of top-100 listed exosomal proteins detected in our supernatants. Even though the overall exosomal protein profile varied between patients, many of these proteins were detected for all or almost all patients. Exosomes influence their target cells through binding to surface receptors, fusion with the cell membrane and subsequent intracellular delivery of molecules, or internalization and subsequent fusion with endosomes followed by transcytosis or lysosomal maturation [75,76]. Exosomes can then influence intracellular processes through delivery of proteins, μRNA and metabolites [77,78,79,80,81,82].

Previous studies of ECM expression at the mRNA level have described a 15-ECM gene expression signature that is associated with survival for patients receiving intensive antileukemic treatment [33,34,35,36]. Ten of the proteins encoded by these 15 genes could also be detected in our present protein studies (Table S8). Our present results at the protein level suggest that the presence of MSCs does not cause any major modulation of this AML-associated prognostic matrix profile.

We investigated 40 consecutive newly diagnosed patient; these patients should therefore be regarded as representative with regard to AML heterogeneity but at the same time they will also be heterogeneous with regard to received antileukemic treatment because approximately half of them will thereby be elderly and/or unfit patients that cannot receive the most intensive treatment (e.g., intensive induction/consolidation, high-dose cytarabine, allogeneic or autologous stem cell transplantation). Due to these differences in intensity and intent (cure versus stabilization) of the antileukemic therapy the patient numbers are too small to allow analysis of differences in survival between various patient subsets. However, due to the higher frequency of elderly patients with secondary AML in the dark yellow cluster in Figure 4, this patient subset will also include a larger fraction of patients not receiving intensive therapy.

Even though AML patients are heterogeneous with regard to extracellular protein release, our study also described several proteins that reach high extracellular levels for all or almost all patients when AML cells were cultured alone (see the Supplementary File 2). Several additional proteins also reached high levels when MSCs were cultured with AML-conditioned medium (Table 4, Table S4). These proteins can be considered as possible therapeutic targets for AML in general, whereas the proteins detected only for (minor) patient subsets should probably be regarded as therapeutic targets in individualized treatment.

Our previous studies have described differences in proteomic profiles between pretreatment AML cells derived from patients that become long-term survivors and patients who later die from chemoresistant relapse despite intensive therapy [46], and between cells isolated at the time of first diagnosis and at the time of chemoresistant relapse for the same patients [55]. We have also described AML cell characteristics that seem to be associated with aging [56]. Our present study, together with a previous [12] study, also suggests that both leukemic and non-leukemic supportive cells contribute to the proteomic profile of the common bone marrow microenvironment. In our opinion, the next step should be to include proteomic analyses as parts of clinical studies to investigate whether these profiles have independent prognostic impact and to possibly allow identification of new single protein biomarkers or therapeutic targets. Such a strategy would require standardized procedures for AML cell sampling and separation/enrichment. One should also try to investigate the possible importance of the extracellular bone marrow microenvironment in these studies. In our opinion, it will be difficult to use in vitro cell culture in routine clinical practice, but proteomic analysis of bone marrow plasma should be an alternative. This would require careful standardization of sampling and preparation of the bone marrow plasma samples, but the advantage with bone marrow plasma would probably be that it reflects the overall contribution of both leukemic and various non-leukemic bone marrow stromal cells to the common extracellular microenvironment. Such approaches used during and following antileukemic therapy have potential to elucidate molecular mechanisms involved in leukemogenesis, chemoresistance and development of relapse from residual leukemic stem cells.

Both conventional intensive chemotherapy and new AML-targeting therapy will probably alter the protein release by the leukemic cells. Previous studies have shown that conventional cytotoxic drugs will also influence various stromal cells (osteoblasts, endothelial cells, immunocompetent cells), including their release of soluble mediators [83,84]. Future studies should therefore try to characterize the effects of various therapeutic strategies on the constitutive protein release by non-leukemic stromal cells.

Targeting of the molecular interactions between malignant cells and their neighboring bone marrow stromal cells is a possible therapeutic strategy in the treatment of cancer [85]. Our present results show that AML patients are very heterogeneous with regard to the leukemic cell contribution to the extracellular matrix. Although the presence of MSCs has only a minor effect on the overall protein release profile by primary AML cells, they reduce patient heterogeneity with regard to extracellular matrix molecules including matrix molecules that are important for the function of the bone marrow stem cell niches. Targeting of the cancer cell/matrix interactions may therefore become possible not only as an individualized therapeutic strategy (i.e., be dependent on patient heterogeneity) but possibly also as a common strategy targeting interactions with bone marrow matrix molecules that are detected for most/all AML patients.

## 5. Conclusions

Primary human AML cells show a wide variation in their constitutive extracellular protein release during in vitro culture both regarding the number of proteins and the function of the released proteins. Bone marrow MSCs also show constitutive protein release especially of extracellular matrix proteins and thereby reduce patient heterogeneity with regard to a minor subset of extracellularly released protein. A large number of exosomal proteins are released both by the AML cells and MSC, suggesting that exosomal release is an important mechanism for extracellular protein release and communication with neighboring cells in the bone marrow microenvironment both for AML cells and MSCs. Therapeutic targeting of exosomal release or molecular interactions between AML cells and the extracellular matrix may represent possible therapeutic strategies in human AML.

## Figures and Tables

**Table 1:** Clinical and biological characteristics of the 40 AML patients included in this study. Unless otherwise stated, the results are presented as the number of patients.

Characteristics (*n* = 40)			
Sex and age		Karyotype/Karyotype Abnormalities	
Males/females	21/19	Normal	20
Age median (range) in years	71 (18–87)	Favorable	4
		Intermediate	9
Predisposition/previous disease		Adverse	4
Previous chronic myeloid neoplasia	1	Not tested	3
Myelodysplastic syndrome	8		
Relapsed AML	3	Flt3 abnormalities	
Chemotherapy related	0	ITD	13
		Wild type	19
Morphology/FAB classification		Not tested	8
M0/M1	17		
M2	8	NPM1 abnormalities	
M4/M5	15	Insertion	13
M6/M7	0	Insertion + Flt3-ITD	8
		Wild type	20
CD34 positive	21	Not tested	7

Abbreviations: FAB, French-American-British; ITD, internal tandem duplication.

**Table 2:** GO analysis of all gene/protein names identified in this study, i.e., all proteins identified for the MSCs cultured alone, 40 primary AML cells cultured alone (AML-CM) and/or MSCs cultured in the presence of AML-conditioned medium (MSC/AML-CM). The presentation is based on a bioinformatical analysis of GO terms/cellular compartment, and the list presents all terms that included at least 100 of the identified proteins (hypergeometric test, Bonferroni correction). The data are presented as the number of proteins associated to a given term, number of proteins in the background dataset, percent of proteins in the dataset annotated to a given GO term, the fold enrichment and the *p*-values.

Cellular Compartment	Number of Proteins	Proteins in the Background Dataset	Percent of Proteins	Fold Enrichment	*p*-Value (Hypergeometric Test)	Bonferroni Corrected
Cytoplasm	1171	5684	68.4	1.8	2.2 × 10^−151^	1.7 × 10^−148^
Nucleus	946	5847	55.3	1.4	2.83 × 10^−41^	2.22 × 10^−38^
Exosomes	746	2043	43.6	3.1	5.8 × 10^−232^	4.5 × 10^−229^
Lysosome	561	1620	32.8	2.9	1.8 × 10^−151^	1.4 × 10^−148^
Nucleolus	443	1257	25.9	3.0	1.4 × 10^−118^	1.1 × 10^−115^
Cytosol	407	1178	23.8	2.9	1.5 × 10^−104^	1.1 × 10^−101^
Mitochondrion	348	1259	20.3	2.4	2.36 × 10^−59^	1.85 × 10^−56^
Centrosome	343	656	20.0	4.4	4.8 × 10^−152^	3.7 × 10^−149^
Plasma membrane	316	3479	18.5	0.8	1	1
Extracellular	262	1825	15.3	1.2	0.000182	0.14294
Endoplasmic reticulum	148	1104	8.6	1.1	0.044427	1
Cytoskeleton	137	427	8.0	2.7	7.52 × 10^−30^	5.89 × 10^−27^
Golgi apparatus	127	897	7.4	1.2	0.013653	1
Nucleoplasm	106	449	6.2	2.0	7.13 × 10^−13^	5.59 × 10^−10^

**Table 3:** Hierarchical clustering of AML patients based on the constitutive protein release profiles of their leukemic cells during in vitro culture. The table gives an overview of the most significant GO terms for each of the five identified protein clusters (see Figure 4, upper part indicating the protein clustering). For each of the five protein clusters, we present the five most significant GO terms (Cellular compartment).

Cluster and Corresponding Go Terms	*p*-Value (Uncorrected)	FDR
**Left purple protein cluster**		
Mitochondrial part	7.77 × 10^−8^	4.68 × 10^−5^
Ribosomal subunit	1.72 × 10^−6^	0.000345
Organelle inner membrane	1.41 × 10^−5^	0.00212
Cytosolic part	1.48 × 10^−5^	0.00212
Mitochondrion	1.86 × 10^−5^	0.00212
**Middle left green protein cluster**		
Nucleoplasm part	7.69 × 10^−7^	0.000557
Nucleoplasm	0.000116	0.0168
Spliceosomal complex	0.000621	0.0749
Nuclear chromosome part	0.00125	0.113
Nucleoplasm part	7.69 × 10^−7^	0.000557
**Middle yellow protein cluster**		
Extracellular exosome	4.72 × 10^−17^	2.97 × 10^−14^
Cytoplasmic vesicle part	1.68 × 10^−9^	1.76 × 10^−7^
Cytosol	1.80 × 10^−9^	1.76 × 10^−7^
Extracellular region	1.42 × 10^−8^	1.11 × 10^−6^
Vesicle lumen	1.42 × 10^−7^	7.43 × 10^−6^
**Middle right orange protein cluster**		
Extracellular matrix organization	2.67 × 10^−10^	1.33 × 10^−6^
Anatomical structure morphogenesis	1.96 × 10^−5^	0.0325
Positive regulation of developmental process	0.000231	0.231
Vesicle-mediated transport	0.000237	0.231
Multicellular organismal process	0.000243	0.231
**Right blue protein cluster**		
No significant GO terms		

**Table 4:** An overview of 60 individual proteins that showed detectable constitutive release by the AML cells for 10 or fewer of the 40 patients (i.e., detected in AML-CM), but showed detectable supernatant levels for at least 30 patients when MSCs were cultured with AML-CM (i.e., MSC/AML-CM). All proteins were quantified in at least five of the seven culture replicates of MSCs alone. The classification is based on information in the Gene database. Proteins that are important for the support of normal hematopoietic stem cells are ***marked*** in the table [28,29,30,31,32].

All identified proteins (alphabetic order)	ABI3BP, B4GALT1, ***BGN***, C1R, CD248, ***CDH2, CDH11***, ***CDH13***, CFH, ***COL10A1***, COL16A1, COL18A1, COL4A1, COL8A1, CRIM1, CTGF, CTHRC1, CTSK, CYR61, DAG1, DKK3, ECM1, ENPP1, ENPP2, FBLN1, FBLN5, FKBP10, GAS6, GOLM1, GREM1, IGFBP4, ISLR, ITGBL1, ***LAMA4***, ***LOX***, ***LOXL1***, ***LOXL2***, MFAP2, MMP13, MMP14, MXRA8, NBL1, NRP2, OLFML2B, PAPPA, PLOD2, PLTP, PROCR, PRSS23, PTPRK, SDC1, ***SMOC1***, SPON2, SRPX2, SSC5D, STC2, TAGLN, THY1, ***TNC***, VASN
Extracellular matrix (ECM) molecules	ABI3BP, ***BGN***, ***COL10A1***, COL16A1, COL18A1, COL4A1, COL8A1, ECM1, FBLN1, FBLN5, ISLR, ***LAMA4***, MFAP2, MXRA8, ***SMOC1***, SPON2, SRPX2, STC2, ***TNC*** ECM modulators: ***LOX***, ***LOXL1***, ***LOXL2***
Cytokines, extracellular soluble mediators	CRIM1 (TGFβ interaction), CTGF, CYR61/CCN1, DKK3 (extracellular Wnt inhibitor), IGFBP4 (IGF binding), PLTP (lipid metabolism),
Cytokine receptors and signaling	ECM1, GAS6, GREM1, NBL1, NRP2, SDC1, SSC5D, VASN (TGF signaling)
Cell surface molecules	Ig superfamily: CD248, THY1 Adhesion: ***CDH2***, ***CDH11***, ***CDH13***, DAG1, ITGBL1 Others: OLFML2B, PROCR, PTPRK, VASN (TGF signaling)
Enzymes	Proteases: C1R, CFH, CTSK, ECM1, MMP13, MMP14, PAPPA, PRSS23 Other enzymes: B4GALT1, ENPP1, ENPP2, ***LOX***, ***LOXL1***, ***LOXL2***
Golgi/endoplasmatic reticulum	B4GALT1, FKBP10 (chaperon), GOLM1
Cytoskeleton	DAG1, TAGLN
Intracellular signaling	CTHRC1, PTPRK

**Table 5:** GO term analyses of the 60 proteins released by a minority of patient samples (≤10) when AML cells were cultured alone but detected for most patient samples (≥30) when MSCs were cultured with AML-conditioned medium (AML-CM). The table presents the significant GO terms (FDR < 0.05) when analyzing Cellular compartments and Molecular functions.

GO Term Identity	Percent Associated Foreground	Percent Associated Background	Fold_Enrichment_Fore- Ground to Background	Foreground_Count	Foreground_n	Background_Count	Background_n	*p*-Value Uncorrected	FDR	Description
**CELLULAR COMPARTMENT**										
GO:0005615	58.3	13.4	4.4	35	60	402	2998	1.29 × 10^−15^	2.67 × 10^−13^	extracellular space
GO:0031012	45.0	8.0	5.6	27	60	240	2998	4.68 × 10^−14^	4.82 × 10^−12^	extracellular matrix
GO:0005576	53.3	17.5	3.0	32	60	525	2998	6.05 × 10^−10^	2.49 × 10^−8^	extracellular region
GO:0044420	20.0	1.8	10.9	12	60	55	2998	2.73 × 10^−9^	9.38 × 10^−8^	extracellular matrix component
GO:0005788	26.6	4.5	5.9	16	60	136	2998	1.43 × 10^−8^	4.21 × 10^−7^	endoplasmic reticulum lumen
GO:0005581	11.6	1.1	10.6	7	60	33	2998	8.81 × 10^−6^	0.000202	collagen trimer
GO:0009986	18.3	4.9	3.8	11	60	146	2998	0.000177	0.00331	cell surface
GO:0005796	8.3	0.9	9.6	5	60	26	2998	0.000282	0.00484	Golgi lumen
GO:0031224	30.0	13.0	2.3	18	60	391	2998	0.000543	0.0086	intrinsic component of membrane
GO:0016323	6.7	0.9	7.4	4	60	27	2998	0.00284	0.0325	basolateral plasma membrane
**MOLECULAR FUNCTION**										
GO:0005509	25.5	5.1	5.0	14	55	152	2968	7.97 × 10^−7^	0.000223	calcium ion binding
GO:0050840	12.7	0.9	14.0	7	55	27	2968	1.64 × 10^−6^	0.00023	extracellular matrix binding
GO:0019838	14.5	1.4	10.0	8	55	43	2968	2.48 × 10^−6^	0.000231	growth factor binding
GO:0005044	7.3	0.4	18.0	4	55	12	2968	0.000152	0.00777	scavenger receptor activity
GO:0005178	10.9	1.7	6.5	6	55	50	2968	0.000443	0.0138	integrin binding
GO:0001968	7.3	0.6	11.4	4	55	19	2968	0.000672	0.0188	fibronectin binding
GO:0016641	5.5	0.2	20.2	3	55	8	2968	0.000848	0.0198	oxidoreductase activity, acting on the CH-NH2 group of donors, oxygen as acceptor
GO:0016015	3.6	0.1	54.0	2	55	2	2968	0.00191	0.0333	morphogen activity
GO:0019955	7.3	0.9	7.7	4	55	28	2968	0.00242	0.0398	cytokine binding
GO:0005201	7.3	1.0	7.4	4	55	29	2968	0.00271	0.042	extracellular matrix structural constituent
GO:0005539	10.9	2.5	4.4	6	55	74	2968	0.00293	0.042	glycosaminoglycan binding
GO:0004528	3.6	0.1	36.0	2	55	3	2968	0.00314	0.042	phosphodiesterase I activity

## Data Availability

The data presented in this study are available on request from the corresponding author.

## Supplementary Materials

The following are available online at https://www.mdpi.com/2072-6694/13/7/1509/s1, Supplementary file 1: Table S1: Characteristics of AML patients included in this study. Table S2: Constitutively MSC-released proteins that are released only by primary AML cells derived from two or fewer of the 40 leukemia patients. Table S3: GO analysis of enriched molecular functions based on all proteins identified in this study, including all proteins identified for MSCs cultured alone, primary AML cells alone (AML-CM) and/or MSCs cultured in the presence of AML-conditioned medium (MSC/AML-CM). Table S4: An overview of proteins showing detectable supernatant levels only for a minority of patients (≤10 patients or 25% of the patients) when AML cells were cultured alone (i.e., AML-conditioned medium) but showing detectable levels for at least 30 patients (i.e., ≥75% of the patients) in MSC cultures prepared with AML-conditioned medium plus fresh medium (1:1 ratio). Table S5: An overview of proteins with significantly increased levels in the supernatants of MSC cultures prepared with AML-conditioned medium compared with the corresponding conditioned medium (Log2 fold change ≥ 1 ≤ −2, *p*-value < 0.05). Table S6: Exosomal proteins detected in the conditioned media after in vitro culture of primary human AML cells. Table S7. Effects of AML-conditioned medium on the constitutive protein release by MSC. Table S8. The 15 ECM signature identified in primary AML cells by gene expression analyses and having a prognostic impact in human AML; a comparison with the results from the present proteomic studies. Figure S1: Protein interaction networks based on the 61 constitutively MSC-released proteins that were detected in ≥4 MSCs and only by ≤2 primary AML cells derived from the 40 leukemia patients. Figure S2: Protein interaction networks based on 144 proteins with significantly different abundance when comparing the AML-CM samples forming cluster 1 and 2 in Figure 4. Figure S3: Identification of AML patient subsets based on their constitutive protein release during in vitro culture; an unsupervised hierarchical cluster analysis including 33 patients. Figure S4: Identification of AML patient subsets based on the protein levels in supernatants derived from MSC cultures supplemented with AML-conditioned medium. Figure S5: A correlation plot comparing the protein expression in seven independent cultures of our MSCs derived from a healthy donor. Supplementary File 2: Quantitative raw data from MaxQuant, processed main data, list of proteins quantified in 50% AML-CM samples and proteins annotated to the GO term Exosome in the present dataset.

## References

[1] Döhner H., Estey E., Grimwade D., Amadori S., Appelbaum F.R., Büchner T., Dombret H., Ebert B.L., Fenaux P., Larson R.A. (2017). Diagnosis and management of AML in adults: 2017 ELN recommendations from an international expert panel. Blood.

[2] Sanz M.A., Fenaux P., Tallman M.S., Estey E.H., Löwenberg B., Naoe T., Lengfelder E., Döhner H., Burnett A.K., Chen S.J. (2019). Management of acute promyelocytic leukemia: Updated recommendations from an expert panel of the European LeukemiaNet. Blood.

[3] Arber D.A., Orazi A., Hasserjian R., Thiele J., Borowitz M.J., Le Beau M.M., Bloomfield C.D., Cazzola M., Vardiman J.W. (2016). The 2016 revision to the World Health Organization classification of myeloid neoplasms and acute leukemia. Blood.

[4] Geyh S., Rodríguez-Paredes M., Jäger P., Khandanpour C., Cadeddu R.P., Gutekunst J., Wilk C.M., Fenk R., Zilkens C., Hermsen D. (2016). Functional inhibition of mesenchymal stromal cells in acute myeloid leukemia. Leukemia.

[5] Bruserud Ø., Aasebø E., Hernandez-Valladares M., Tsykunova G., Reikvam H. (2017). Therapeutic targeting of leukemic stem cells in acute myeloid leukemia–the biological background for possible strategies. Expert Opin. Drug Discov..

[6] Brenner A.K., Nepstad I., Bruserud Ø. (2017). Mesenchymal Stem Cells Support Survival and Proliferation of Primary Human Acute Myeloid Leukemia Cells through Heterogeneous Molecular Mechanisms. Front. Immunol..

[7] Ehninger A., Trumpp A. (2011). The bone marrow stem cell niche grows up: Mesenchymal stem cells and macrophages move in. J. Exp. Med..

[8] Brenner A.K., Tvedt T.H., Nepstad I., Rye K.P., Hagen K.M., Reikvam H., Bruserud Ø. (2017). Patients with acute myeloid leukemia can be subclassified based on the constitutive cytokine release of the leukemic cells; the possible clinical relevance and the importance of cellular iron metabolism. Expert Opin. Ther. Targets.

[9] Bruserud Ø., Gjertsen B.T., Foss B., Huang T.S. (2001). New strategies in the treatment of acute myelogenous leukemia (AML): In vitro culture of aml cells--the present use in experimental studies and the possible importance for future therapeutic approaches. Stem Cells.

[10] Eltoukhy H.S., Sinha G., Moore C.A., Gergues M., Rameshwar P. (2018). Secretome within the bone marrow microenvironment: A basis for mesenchymal stem cell treatment and role in cancer dormancy. Biochimie.

[11] Kaushansky K., Zhan H. (2018). The regulation of normal and neoplastic hematopoiesis is dependent on microenvironmental cells. Adv. Biol. Regul..

[12] Aasebø E., Birkeland E., Selheim F., Berven F., Brenner A.K., Bruserud Ø. (2020). The Extracellular Bone Marrow Microenvironment-A Proteomic Comparison of Constitutive Protein Release by In Vitro Cultured Osteoblasts and Mesenchymal Stem Cells. Cancers.

[13] Cox J., Mann M. (2008). MaxQuant enables high peptide identification rates, individualized p.p.b.-range mass accuracies and proteome-wide protein quantification. Nat. Biotechnol..

[14] Cox J., Matic I., Hilger M., Nagaraj N., Selbach M., Olsen J.V., Mann M. (2009). A practical guide to the MaxQuant computational platform for SILAC-based quantitative proteomics. Nat. Protoc..

[15] Tyanova S., Temu T., Sinitcyn P., Carlson A., Hein M.Y., Geiger T., Mann M., Cox J. (2016). The Perseus computational platform for comprehensive analysis of (prote)omics data. Nat. Methods.

[16] Pathan M., Keerthikumar S., Ang C.S., Gangoda L., Quek C.Y., Williamson N.A., Mouradov D., Sieber O.M., Simpson R.J., Salim A. (2015). FunRich: An open access standalone functional enrichment and interaction network analysis tool. Proteomics.

[17] Scholz C., Lyon D., Refsgaard J.C., Jensen L.J., Choudhary C., Weinert B.T. (2015). Avoiding abundance bias in the functional annotation of post-translationally modified proteins. Nat. Methods.

[18] Arntzen M.Ø., Koehler C.J., Barsnes H., Berven F.S., Treumann A., Thiede B. (2011). IsobariQ: Software for isobaric quantitative proteomics using IPTL, iTRAQ, and TMT. J. Proteome Res..

[19] Szklarczyk D., Morris J.H., Cook H., Kuhn M., Wyder S., Simonovic M., Santos A., Doncheva N.T., Roth A., Bork P. (2017). The STRING database in 2017: Quality-controlled protein-protein association networks, made broadly accessible. Nucleic Acids Res..

[20] Shannon P., Markiel A., Ozier O., Baliga N.S., Wang J.T., Ramage D., Amin N., Schwikowski B., Ideker T. (2003). Cytoscape: A software environment for integrated models of biomolecular interaction networks. Genome Res..

[21] Bader G.D., Hogue C.W. (2003). An automated method for finding molecular complexes in large protein interaction networks. BMC Bioinform..

[22] Brenner A.K., Aasebø E., Hernandez-Valladares M., Selheim F., Berven F., Grønningsæter I.S., Bartaula-Brevik S., Bruserud Ø. (2019). The Capacity of Long-Term in Vitro Proliferation of Acute Myeloid Leukemia Cells Supported Only by Exogenous Cytokines Is Associated with a Patient Subset with Adverse Outcome. Cancers.

[23] Ryningen A., Ersvaer E., Øyan A.M., Kalland K.H., Vintermyr O.K., Gjertsen B.T., Bruserud Ø. (2006). Stress-induced in vitro apoptosis of native human acute myelogenous leukemia (AML) cells shows a wide variation between patients and is associated with low BCL-2:Bax ratio and low levels of heat shock protein 70 and 90. Leuk. Res..

[24] Reikvam H., Aasebø E., Brenner A.K., Bartaula-Brevik S., Grønningsæter I.S., Forthun R.B., Hovland R., Bruserud Ø. (2019). High Constitutive Cytokine Release by Primary Human Acute Myeloid Leukemia Cells Is Associated with a Specific Intercellular Communication Phenotype. J. Clin. Med..

[25] Honnemyr M., Bruserud Ø., Brenner A.K. (2017). The constitutive protease release by primary human acute myeloid leukemia cells. J. Cancer Res. Clin. Oncol..

[26] Bruserud Ø. (1996). Effects of endogenous interleukin 1 on blast cells derived from acute myelogenous leukemia patients. Leuk. Res..

[27] Bruserud Ø., Ryningen A., Olsnes A.M., Stordrange L., Øyan A.M., Kalland K.H., Gjertsen B.T. (2007). Subclassification of patients with acute myelogenous leukemia based on chemokine responsiveness and constitutive chemokine release by their leukemic cells. Haematologica.

[28] Domingues M.J., Cao H., Heazlewood S.Y., Cao B., Nilsson S.K. (2017). Niche Extracellular Matrix Components and Their Influence on HSC. J. Cell Biochem..

[29] Sangaletti S., Chiodoni C., Tripodo C., Colombo M.P. (2017). Common extracellular matrix regulation of myeloid cell activity in the bone marrow and tumor microenvironments. Cancer Immunol. Immunother..

[30] Klamer S., Voermans C. (2014). The role of novel and known extracellular matrix and adhesion molecules in the homeostatic and regenerative bone marrow microenvironment. Cell Adh. Migr..

[31] Vedi A., Santoro A., Dunant C.F., Dick J.E., Laurenti E. (2016). Molecular landscapes of human hematopoietic stem cells in health and leukemia. Ann. N. Y. Acad. Sci..

[32] Yu V.W., Scadden D.T. (2016). Hematopoietic Stem Cell and Its Bone Marrow Niche. Curr. Top. Dev. Biol..

[33] Lee D., Kim D.W., Cho J.Y. (2020). Role of growth factors in hematopoietic stem cell niche. Cell Biol. Toxicol..

[34] Izzi V., Lakkala J., Devarajan R., Savolainen E.R., Koistinen P., Heljasvaara R., Pihlajaniemi T. (2018). Vanin 1 (VNN1) levels predict poor outcome in acute myeloid leukemia. Am. J. Hematol..

[35] Izzi V., Lakkala J., Devarajan R., Savolainen E.R., Koistinen P., Heljasvaara R., Pihlajaniemi T. (2017). Expression of a specific extracellular matrix signature is a favorable prognostic factor in acute myeloid leukemia. Leuk. Res. Rep..

[36] Izzi V., Lakkala J., Devarajan R., Ruotsalainen H., Savolainen E.R., Koistinen P., Heljasvaara R., Pihlajaniemi T. (2017). An extracellular matrix signature in leukemia precursor cells and acute myeloid leukemia. Haematologica.

[37] Takahashi K., Eto H., Tanabe K.K. (1999). Involvement of CD44 in matrix metalloproteinase-2 regulation in human melanoma cells. Int. J. Cancer.

[38] Ponta H., Sherman L., Herrlich P.A. (2003). CD44: From adhesion molecules to signalling regulators. Nat. Rev. Mol. Cell Biol..

[39] Klingbeil P., Marhaba R., Jung T., Kirmse R., Ludwig T., Zöller M. (2009). CD44 variant isoforms promote metastasis formation by a tumor cell-matrix cross-talk that supports adhesion and apoptosis resistance. Mol. Cancer Res..

[40] Schuurhuis G.J., Meel M.H., Wouters F., Min L.A., Terwijn M., de Jonge N.A., Kelder A., Snel A.N., Zweegman S., Ossenkoppele G.J. (2013). Normal hematopoietic stem cells within the AML bone marrow have a distinct and higher ALDH activity level than co-existing leukemic stem cells. PLoS ONE.

[41] Ishii S., Ford R., Thomas P., Nachman A., Steele G., Jessup J.M. (1993). CD44 participates in the adhesion of human colorectal carcinoma cells to laminin and type IV collagen. Surg. Oncol..

[42] Spertini C., Baïsse B., Bellone M., Gikic M., Smirnova T., Spertini O. (2019). Acute Myeloid and Lymphoblastic Leukemia Cell Interactions with Endothelial Selectins: Critical Role of PSGL-1, CD44 and CD43. Cancers.

[43] Winkler I.G., Barbier V., Nowlan B., Jacobsen R.N., Forristal C.E., Patton J.T., Magnani J.L., Lévesque J.P. (2012). Vascular niche E-selectin regulates hematopoietic stem cell dormancy, self renewal and chemoresistance. Nat. Med..

[44] Yokota A., Ishii G., Sugaya Y., Nishimura M., Saito Y., Harigaya K. (1998). Expression of exon v6-containing CD44 isoforms is related to poor prognosis of acute myelocytic leukemia. Hematol. Oncol..

[45] Legras S., Günthert U., Stauder R., Curt F., Oliferenko S., Kluin-Nelemans H.C., Marie J.P., Proctor S., Jasmin C., Smadja-Joffe F. (1998). A strong expression of CD44-6v correlates with shorter survival of patients with acute myeloid leukemia. Blood.

[46] Aasebø E., Berven F.S., Bartaula-Brevik S., Stokowy T., Hovland R., Vaudel M., Døskeland S.O., McCormack E., Batth T.S., Olsen J.V. (2020). Proteome and Phosphoproteome Changes Associated with Prognosis in Acute Myeloid Leukemia. Cancers.

[47] Gjertsen B.T., Øyan A.M., Marzolf B., Hovland R., Gausdal G., Døskeland S.O., Dimitrov K., Golden A., Kalland K.H., Hood L. (2002). Analysis of acute myelogenous leukemia: Preparation of samples for genomic and proteomic analyses. J. Hematother. Stem Cell Res..

[48] Wheatley K., Burnett A.K., Goldstone A.H., Gray R.G., Hann I.M., Harrison C.J., Rees J.K., Stevens R.F., Walker H.A. (1999). Simple, robust, validated and highly predictive index for the determination of risk-directed therapy in acute myeloid leukaemia derived from the MRC AML 10 trial. United Kingdom Medical Research Council’s Adult and Childhood Leukaemia Working Parties. Br. J. Haematol..

[49] de Jonge H.J., Valk P.J., de Bont E.S., Schuringa J.J., Ossenkoppele G., Vellenga E., Huls G. (2011). Prognostic impact of white blood cell count in intermediate risk acute myeloid leukemia: Relevance of mutated NPM1 and FLT3-ITD. Haematologica.

[50] Lin P., Chen L., Luthra R., Konoplev S.N., Wang X., Medeiros L.J. (2008). Acute myeloid leukemia harboring t(8;21)(q22;q22): A heterogeneous disease with poor outcome in a subset of patients unrelated to secondary cytogenetic aberrations. Mod. Pathol..

[51] Feng S., Zhou L., Zhang X., Tang B., Zhu X., Liu H., Sun Z., Zheng C. (2019). Impact Of ELN Risk Stratification, Induction Chemotherapy Regimens And Hematopoietic Stem Cell Transplantation On Outcomes In Hyperleukocytic Acute Myeloid Leukemia With Initial White Blood Cell Count More Than 100 × 10^9^/L. Cancer Manag. Res..

[52] How J., Sykes J., Gupta V., Yee K.W., Schimmer A.D., Schuh A.C., Minden M.D., Kamel-Reid S., Brandwein J.M. (2012). Influence of FLT3-internal tandem duplication allele burden and white blood cell count on the outcome in patients with intermediate-risk karyotype acute myeloid leukemia. Cancer.

[53] Thomas D., Majeti R. (2017). Biology and relevance of human acute myeloid leukemia stem cells. Blood.

[54] Hernandez-Valladares M., Bruserud Ø., Selheim F. (2020). The Implementation of Mass Spectrometry-Based Proteomics Workflows in Clinical Routines of Acute Myeloid Leukemia: Applicability and Perspectives. Int. J. Mol. Sci..

[55] Aasebø E., Berven F.S., Hovland R., Døskeland S.O., Bruserud Ø., Selheim F., Hernandez-Valladares M. (2020). The Progression of Acute Myeloid Leukemia from First Diagnosis to Chemoresistant Relapse: A Comparison of Proteomic and Phosphoproteomic Profiles. Cancers.

[56] Hernandez-Valladares M., Aasebø E., Berven F., Selheim F., Bruserud Ø. (2020). Biological characteristics of aging in human acute myeloid leukemia cells: The possible importance of aldehyde dehydrogenase, the cytoskeleton and altered transcriptional regulation. Aging.

[57] Mer A.S., Lindberg J., Nilsson C., Klevebring D., Wang M., Grönberg H., Lehmann S., Rantalainen M. (2018). Expression levels of long non-coding RNAs are prognostic for AML outcome. J. Hematol. Oncol..

[58] Stäubert C., Bhuiyan H., Lindahl A., Broom O.J., Zhu Y., Islam S., Linnarsson S., Lehtiö J., Nordström A. (2015). Rewired metabolism in drug-resistant leukemia cells: A metabolic switch hallmarked by reduced dependence on exogenous glutamine. J. Biol. Chem..

[59] Lazarevic V., Hörstedt A.S., Johansson B., Antunovic P., Billström R., Derolf Å., Lehmann S., Möllgård L., Peterson S., Stockelberg D. (2015). Failure matters: Unsuccessful cytogenetics and unperformed cytogenetics are associated with a poor prognosis in a population-based series of acute myeloid leukaemia. Eur. J. Haematol..

[60] Grønbæk K., Müller-Tidow C., Perini G., Lehmann S., Bach Treppendahl M., Mills K., Plass C., Schlegelberger B., European Genomics and Epigenomics Study on MDS and AML (EuGESMA), COST Action BM0801 (2012). A critical appraisal of tools available for monitoring epigenetic changes in clinical samples from patients with myeloid malignancies. Haematologica.

[61] Eppert K., Takenaka K., Lechman E.R., Waldron L., Nilsson B., van Galen P., Metzeler K.H., Poeppl A., Ling V., Beyene J. (2011). Stem cell gene expression programs influence clinical outcome in human leukemia. Nat. Med..

[62] Malhotra V., Erlmann P. (2015). The pathway of collagen secretion. Annu. Rev. Cell Dev. Biol..

[63] Granfeldt Østgård L.S., Medeiros B.C., Sengeløv H., Nørgaard M., Andersen M.K., Dufva I.H., Friis L.S., Kjeldsen E., Marcher C.W., Preiss B. (2015). Epidemiology and Clinical Significance of Secondary and Therapy-Related Acute Myeloid Leukemia: A National Population-Based Cohort Study. J. Clin. Oncol..

[64] Hulegårdh E., Nilsson C., Lazarevic V., Garelius H., Antunovic P., Rangert Derolf Å., Möllgård L., Uggla B., Wennström L., Wahlin A. (2015). Characterization and prognostic features of secondary acute myeloid leukemia in a population-based setting: A report from the Swedish Acute Leukemia Registry. Am. J. Hematol..

[65] Hatfield K.J., Reikvam H., Bruserud Ø. (2010). The crosstalk between the matrix metalloprotease system and the chemokine network in acute myeloid leukemia. Curr. Med. Chem..

[66] Afonina I.S., Müller C., Martin S.J., Beyaert R. (2015). Proteolytic Processing of Interleukin-1 Family Cytokines: Variations on a Common Theme. Immunity.

[67] Bruserud Ø., Aasen I., Akselsen P.E., Bergheim J., Rasmussen G., Nesthus I. (1996). Interleukin 1 receptor antagonist (IL1RA) in acute leukaemia: IL1RA is both secreted spontaneously by myelogenous leukaemia blasts and is a part of the acute phase reaction in patients with chemotherapy-induced leucopenia. Eur. J. Haematol..

[68] Fukushima T., Uchiyama S., Tanaka H., Kataoka H. (2018). Hepatocyte Growth Factor Activator: A Proteinase Linking Tissue Injury with Repair. Int. J. Mol. Sci..

[69] Brenner A.K., Bruserud Ø. (2019). Functional Toll-Like Receptors (TLRs) Are Expressed by a Majority of Primary Human Acute Myeloid Leukemia Cells and Inducibility of the TLR Signaling Pathway Is Associated with a More Favorable Phenotype. Cancers.

[70] Wensink A.C., Hack C.E., Bovenschen N. (2015). Granzymes regulate proinflammatory cytokine responses. J. Immunol..

[71] Baschuk N., Wang N., Watt S.V., Halse H., House C., Bird P.I., Strugnell R., Trapani J.A., Smyth M.J., Andrews D.M. (2014). NK cell intrinsic regulation of MIP-1α by granzyme M. Cell Death Dis..

[72] Lee S.E., Jeong S.K., Lee S.H. (2010). Protease and protease-activated receptor-2 signaling in the pathogenesis of atopic dermatitis. Yonsei Med. J..

[73] Reiss K., Saftig P. (2009). The "a disintegrin and metalloprotease" (ADAM) family of sheddases: Physiological and cellular functions. Semin. Cell Dev. Biol..

[74] Qureshi N., Morrison D.C., Reis J. (2012). Proteasome protease mediated regulation of cytokine induction and inflammation. Biochim. Biophys. Acta.

[75] Deng F., Miller J. (2019). A review on protein markers of exosome from different bio- resources and the antibodies used for characterization. J. Histotechnol..

[76] Pegtel D.M., Gould S.J. (2019). Exosomes. Annu. Rev. Biochem..

[77] Zhang J., Li S., Li L., Li M., Guo C., Yao J., Mi S. (2015). Exosome and exosomal microRNA: Trafficking, sorting, and function. Genom. Proteom. Bioinform..

[78] Kalluri R., LeBleu V.S. (2020). The biology, function, and biomedical applications of exosomes. Science.

[79] Kreitz J., Schönfeld C., Seibert M., Stolp V., Alshamleh I., Oellerich T., Steffen B., Schwalbe H., Schnütgen F., Kurrle N. (2019). Metabolic Plasticity of Acute Myeloid Leukemia. Cells.

[80] Castro I., Sampaio-Marques B., Ludovico P. (2019). Targeting Metabolic Reprogramming in Acute Myeloid Leukemia. Cells.

[81] Jones C.L., Stevens B.M., D’Alessandro A., Reisz J.A., Culp-Hill R., Nemkov T., Pei S., Khan N., Adane B., Ye H. (2018). Inhibition of Amino Acid Metabolism Selectively Targets Human Leukemia Stem Cells. Cancer Cell.

[82] Chen W.L., Wang J.H., Zhao A.H., Xu X., Wang Y.H., Chen T.L., Li J.M., Mi J.Q., Zhu Y.M., Liu Y.F. (2014). A distinct glucose metabolism signature of acute myeloid leukemia with prognostic value. Blood.

[83] Ersvaer E., Brenner A.K., Vetås K., Reikvam H., Bruserud Ø. (2015). Effects of cytarabine on activation of human T cells-cytarabine has concentration-dependent effects that are modulated both by valproic acid and all-trans retinoic acid. BMC Pharmacol. Toxicol..

[84] Kvestad H., Evensen L., Lorens J.B., Bruserud O., Hatfield K.J. (2014). In Vitro Characterization of Valproic Acid, ATRA, and Cytarabine Used for Disease-Stabilization in Human Acute Myeloid Leukemia: Antiproliferative Effects of Drugs on Endothelial and Osteoblastic Cells and Altered Release of Angioregulatory Mediators by Endothelial Cells. Leuk. Res. Treatmen.

[85] Zheng H., Bae Y., Kasimir-Bauer S., Tang R., Chen J., Ren G., Yuan M., Esposito M., Li W., Wei Y. (2017). Therapeutic Antibody Targeting Tumor- and Osteoblastic Niche-Derived Jagged1 Sensitizes Bone Metastasis to Chemotherapy. Cancer Cell.

