# Supplementary material for: The Constitutive Extracellular Protein Release by Acute Myeloid Leukemia Cells—A Proteomic Study of Patient Heterogeneity and Its Modulation by Mesenchymal Stromal Cells
Source: Cancers (Basel). 2021 Mar 25;13(7):1509. doi: 10.3390/cancers13071509 (PMC8037744; doi:10.3390/cancers13071509)
Supplement: Supplementary file 1 [file cancers-13-01509-s001.zip › cancers-1148043-supplementary-update/Supplementary file 1.docx]

**The Constitutive Extracellular Protein Release by Acute Myeloid Leukemia Cells—A Proteomic Study of Patient Heterogeneity and Its Modulation by Mesenchymal Stromal Cells**

**Elise Aasebø, Annette K. Brenner, Even Birkeland, Tor Henrik Anderson Tvedt, Frode Selheim,**

**Frode S. Berven and Øystein Bruserud**

**Table S1.** Clinical and biological characteristics of AML patients included in the study. The patients are listed according to the two main patient clusters (yellow and brown color respectively) identified in Figure 2. Each of these two main clusters could be further classified into two subclusters (bright/dark color). Survival is given in months and is included only for those patients receiving intensive and potential curative treatment; for the other patients we state "No treatment" and this term means no such intensive treatment. .

| **Patient Code** | **Gender** | **Age** | **Other** | **FAB** | **Cytogenetics** | **FLT3 Status** | **NPM1 Status** | **CD34+ Status** | **Proliferation (3H-tymidin)** | **Viability (%)** | **Survival (Jan 2018)** |
| --- | --- | --- | --- | --- | --- | --- | --- | --- | --- | --- | --- |
| P115 | F | 87 |  | M0 | del(5) | wt | wt | pos | 1597 | 8.2 | no treatment |
| P15 | F | 67 | Relapsed AML | M0 | +21 | wt | wt | pos | 542 | 47.8 | 7 |
| VA7 | M | 71 |  | M4/5 | nt | nt | nt | na | 477 | 52.7 | no treatment |
| P1 | M | 42 |  | M2 | Normal | ITD | wt | hetero | 335 | 9 | >82 |
| P205 | F | 55 |  | M2 | normal | ITD | ins | neg | 319 | 46.1 | 24 |
| P218 | F | 77 |  | M1 | nt | nt | ins | neg | 782 | 18.7 | no treatment |
| P155 | F | 71 |  | M0 | Normal | wt | ins | neg | 552 | 8 | no treatment |
| P213 | F | 79 |  |  | Normal | ITD | ins | hetero | 5382 | 35.9 | no treatment |
| P210 | M | 78 |  | M1 | Multiple | nt | nt | pos | 1175 | 39.1 | no treatment |
| P151 | F | 71 | MDS, now AML relapse |  | del(12) | nt | nt | pos | 4664 | 70.9 | 26 |
| P206 | M | 76 | MDS 6 months |  | Normal | nt | nt | pos | 497 | 8 | no treatment |
| P127 | M | 41 |  | M1 | t(8;21) del9-20 -22 -3mar | wt | wt | pos | 550 | 0.5 | unknown |
| P214 | M | 72 | MDS | M4 | Normal | nt | nt | neg | 401 | 6.2 | no treatment |
| VA14 | F | 77 | MDS 2 years | M1 | Normal | wt | wt | pos | 1168 | 83.6 | no treatment |
| P200 | M | 79 | MDS 2 years | M2 | +8, -9, +mar | wt | wt | pos | 2487 | 68.9 | no treatment |
| P201 | M | 78 | CMML 9 months | M4 | +8 | nt | nt | neg | 217 | 3.3 | no treatment |
| P211 | M | 87 | Myelofibrosis | M1 | del(20) | wt | wt | pos | 1386 | 18.4 | no treatment |
| P116 | M | 36 |  | M5 | +8, +22, inv16 | ITD | wt | pos | 982 | 43.1 | unknown |
| P209 | M | 82 |  |  | +8 | wt | wt | pos | 253 | 4.9 | no treatment |
| P152 | F | 72 | Relapsed AML | M2 | Normal | ITD | ins | pos | 826 | 7 | 3 |
| P216 | M | 59 | CMML | M5 | del(20), +8 | ITD | wt | pos | 362 | 9.8 | unknown |
| VA3 | F | 78 |  | M1 | Normal | ITD | ins | neg | 5212 | 13.7 | no treatment |
| P9 | M | 46 |  | M1 | Normal | wt | ins | na | 841 | 46.2 | 26 |
| P204 | F | 66 |  | M4/5 | t(9;15) | ITD | wt | neg | 385 | 3 | 7 |
| P217 | F | 46 |  | M2 | inv(13) | wt | wt | pos | 3241 | 70.7 | 29 |
| P207 | F | 74 | CMML | M5 | Normal | ITD | ins | neg | 1830 | 36.1 | no treatment |
| P215 | F | 55 |  | M1 | Normal | ITD | ins | hetero | 6288 | 29.7 | 8 |
| VA5 | F | 77 | Possible MDS | M1/2 | Normal | ITD | ins | na | 13769 | 54.4 | no treatment |
| P208 | M | 78 |  | M1 | Normal | wt | wt | pos | 338 | 23 | no treatment |
| P40 | F | 63 |  | M1 | Normal | wt | wt | pos | 224 | 26.1 | 5 |
| P11 | F | 18 |  | M4 | inv16 | wt | wt | pos | 1878 | 55.2 | > 84 |
| P38 | M | 20 |  | M2 | Normal | ITD | wt | pos | 10582 | 57 | 27 |
| P120 | F | 68 |  | M5 | Normal | wt | ins | neg | 5240 | 14.4 | 1 (toxic) |
| P4 | M | 60 |  | M5 | t(10;11), +8 | wt | wt | na | 384 | 20.3 | 16 |
| P219 | M | 19 |  | M5 | Normal | wt | wt | neg | 637 | 11.7 | > 30 |
| P34 | M | 48 |  | M5 | Normal | ITD | ins | na | 25887 | 59.7 | 7 |
| P37 | F | 57 |  | M4 | inv16 | wt | wt | pos | 6761 | 55.4 | 14 (denied further treatment) |
| P124 | M | 76 | CMML | M5 | del(12), -7 | nt | nt | pos | 4263 | 11.2 | 6 |
| P125 | M | 64 |  | M5 | Normal | wt | ins | neg | 1285 | 30.8 | 1 (toxic) |
| P203 | M | 83 |  | M1 | nt | wt | wt | pos | 4158 | 7.3 | no treatment |

**Table S2.** Constitutively MSC-released proteins that are detected in more than four MSC supernatants and only in conditioned media from primary AML cells derived from two or less of the 40 leukemia patients. The table lists the proteins and their inclusion into the GO terms Soluble cytokine receptors, Cytokine, Protease, Exosome, Soluble adhesion molecules and Extracellular matrix molecules. Proteins not detected for any AML patient are marked with grey shadow.

| **Gene Name** | **Protein Name** | **Detected in #MSC** | **Detected in #AML-CM** | **Soluble Cytokine receptor** | **Cytokine** | **Protease** | **Exosome** | **Soluble Adhesion Molecules** | **Extracellular Matrix Molecule** |
| --- | --- | --- | --- | --- | --- | --- | --- | --- | --- |
| ADAMTS1 | A disintegrin and metalloproteinase with thrombospondin motifs 1 | 5 | 1 |  |  |  |  |  |  |
| ADAMTSL1 | ADAMTS-like protein 1 | 6 | 1 |  |  |  |  |  |  |
| ANGPTL4 | Angiopoietin-related protein 4 | 7 | 0 |  |  |  |  |  |  |
| ANTXR1 | Anthrax toxin receptor 1 | 5 | 0 |  |  |  |  |  |  |
| C1QB | Complement C1q subcomponent subunit B | 4 | 1 |  |  |  |  |  |  |
| CAV1 | Caveolin-1 | 5 | 0 |  |  |  |  |  |  |
| CCBE1 | Collagen and calcium-binding EGF domain-containing protein 1 | 4 | 1 |  |  |  |  |  |  |
| CLMP | CXADR-like membrane protein | 4 | 1 |  |  |  |  |  |  |
| CNN3 | Calponin-3 | 5 | 1 |  |  |  |  |  |  |
| COL10A1 | Collagen alpha-1(X) chain | 7 | 2 |  |  |  |  |  |  |
| COL16A1 | Collagen alpha-1(XVI) chain | 7 | 1 |  |  |  |  |  |  |
| COL6A2 | Collagen alpha-2(VI) chain | 5 | 2 |  |  |  |  |  |  |
| COL6A3 | Collagen alpha-3(VI) chain | 4 | 2 |  |  |  |  |  |  |
| CPE | Carboxypeptidase E | 5 | 2 |  |  |  |  |  |  |
| CTSK | Cathepsin K | 6 | 1 |  |  |  |  |  |  |
| DCBLD2 | Discoidin, CUB and LCCL domain-containing protein 2 | 7 | 0 |  |  |  |  |  |  |
| DNAJC3 | DnaJ homolog subfamily C member 3 | 6 | 2 |  |  |  |  |  |  |
| EDIL3 | EGF-like repeat and discoidin I-like domain-containing protein 3 | 5 | 1 |  |  |  |  |  |  |
| ENG | Endoglin | 5 | 2 |  |  |  |  |  |  |
| EXT2 | Exostosin-2 | 6 | 2 |  |  |  |  |  |  |
| EXTL2 | Exostosin-like 2;Processed exostosin-like 2 | 5 | 1 |  |  |  |  |  |  |
| FAP | Prolyl endopeptidase FAP;Antiplasmin-cleaving enzyme FAP, soluble form | 7 | 2 |  |  |  |  |  |  |
| FBN2 | Fibrillin-2 | 6 | 0 |  |  |  |  |  |  |
| FLRT2 | Leucine-rich repeat transmembrane protein FLRT2 | 4 | 1 |  |  |  |  |  |  |
| FN1 | Fibronectin;Anastellin;Ugl-Y1;Ugl-Y2;Ugl-Y3 | 6 | 2 |  |  |  |  |  |  |
| FST | Follistatin | 6 | 2 |  |  |  |  |  |  |
| FSTL3 | Follistatin-related protein 3 | 5 | 0 |  |  |  |  |  |  |
| GALNT5 | Polypeptide N-acetylgalactosaminyltransferase 5 | 6 | 2 |  |  |  |  |  |  |
| GNG12 | Guanine nucleotide-binding protein G(I)/G(S)/G(O) subunit gamma-12 | 5 | 1 |  |  |  |  |  |  |
| HMCN1 | Hemicentin-1 | 5 | 2 |  |  |  |  |  |  |
| LOXL1 | Lysyl oxidase homolog 1 | 7 | 1 |  |  |  |  |  |  |
| LOXL3 | Lysyl oxidase homolog 3 | 6 | 2 |  |  |  |  |  |  |
| LRRC15 | Leucine-rich repeat-containing protein 15 | 4 | 0 |  |  |  |  |  |  |
| LTBP3 | Latent-transforming growth factor beta-binding protein 3 | 5 | 1 |  |  |  |  |  |  |
| MAK16 | Protein MAK16 homolog | 6 | 2 |  |  |  |  |  |  |
| MAN1B1 | Endoplasmic reticulum mannosyl-oligosaccharide 1,2-alpha-mannosidase | 7 | 1 |  |  |  |  |  |  |
| MCAM | Cell surface glycoprotein MUC18 | 4 | 1 |  |  |  |  |  |  |
| MFAP2 | Microfibrillar-associated protein 2 | 7 | 0 |  |  |  |  |  |  |
| MFAP4 | Microfibril-associated glycoprotein 4 | 4 | 0 |  |  |  |  |  |  |
| MXRA7 | Matrix-remodeling-associated protein 7 | 4 | 2 |  |  |  |  |  |  |
| NTM | Neurotrimin | 7 | 2 |  |  |  |  |  |  |
| PAM | Peptidyl-glycine alpha-amidating monooxygenase;Peptidylglycine alpha-hydroxylating monooxygenase;Peptidyl-alpha-hydroxyglycine alpha-amidating lyase | 5 | 2 |  |  |  |  |  |  |
| PBRM1 | Protein polybromo-1 | 5 | 0 |  |  |  |  |  |  |
| PCDH9 | Protocadherin-9 | 5 | 0 |  |  |  |  |  |  |
| PDGFRB | Platelet-derived growth factor receptor beta | 4 | 1 |  |  |  |  |  |  |
| PLAT | Tissue-type plasminogen activator;Tissue-type plasminogen activator chain A;Tissue-type plasminogen activator chain B | 4 | 0 |  |  |  |  |  |  |
| PLOD2 | Procollagen-lysine,2-oxoglutarate 5-dioxygenase 2 | 7 | 1 |  |  |  |  |  |  |
| POSTN | Periostin | 5 | 1 |  |  |  |  |  |  |
| POSTN | Periostin | 6 | 2 |  |  |  |  |  |  |
| POSTN | Periostin | 4 | 0 |  |  |  |  |  |  |
| PRG4 | Proteoglycan 4;Proteoglycan 4 C-terminal part | 7 | 0 |  |  |  |  |  |  |
| PRKCDBP | Protein kinase C delta-binding protein | 5 | 0 |  |  |  |  |  |  |
| PRSS23 | Serine protease 23 | 7 | 2 |  |  |  |  |  |  |
| S100A13 | Protein S100-A13 | 7 | 1 |  |  |  |  |  |  |
| SDC1 | Syndecan-1 | 7 | 1 |  |  |  |  |  |  |
| SERPINA3 | Alpha-1-antichymotrypsin;Alpha-1-antichymotrypsin His-Pro-less | 6 | 2 |  |  |  |  |  |  |
| STC1 | Stanniocalcin-1 | 4 | 2 |  |  |  |  |  |  |
| TAPBP | Tapasin | 7 | 1 |  |  |  |  |  |  |
| THY1 | Thy-1 membrane glycoprotein | 7 | 0 |  |  |  |  |  |  |
| TSPAN4 | Tetraspanin-4 | 4 | 2 |  |  |  |  |  |  |
| ULBP2 | NKG2D ligand 2 | 7 | 1 |  |  |  |  |  |  |

**Table S3.** GO analysis of the gene names representing all proteins identified in the study, including all proteins identified for MSCs cultured alone, primary AML cells alone (AML-CM) and/or MSCs cultured in the presence of AML conditioned medium (MSC/AML-CM). The presentation is based on a bioinformatical analysis of GO terms/molecular function, and the list includes all terms showing a significant p-value (hypergeometric test, Bonferroni correction). The data are presented as the number of proteins associated to a given term, number of proteins in the background dataset, percent of proteins in the dataset annotated to a given GO term, the fold enrichment and the *p*-values.

| **Molecular Function** | **Number of Proteins** | **Proteins in the Background Dataset** | **Percentage of Proteins** | **Fold Enrichment** | ***p*-value (Hypergeometric Test)** | **Bonferroni Correction** |
| --- | --- | --- | --- | --- | --- | --- |
| RNA binding | 111 | 366 | 6.3 | 3.1 | 8.22 × 10^−29^ | 1.84 × 10^−26^ |
| Structural constituent of ribosome | 66 | 152 | 3.7 | 4.5 | 1.35 × 10^−27^ | 3.02 × 10^−25^ |
| Chaperone activity | 50 | 126 | 2.8 | 4.1 | 3.56 × 10^−19^ | 7.98 × 10^−17^ |
| Translation regulator activity | 40 | 101 | 2.3 | 4.1 | 1.39 × 10^−15^ | 3.11 × 10^−13^ |
| Isomerase activity | 24 | 45 | 1.4 | 5.5 | 2.3 × 10^−13^ | 5.15 × 10^−11^ |
| Ribonucleoprotein | 21 | 41 | 1.2 | 5.3 | 2.04 × 10^−11^ | 4.56 × 10^−09^ |
| Catalytic activity | 96 | 532 | 5.4 | 1.9 | 1.73 × 10^−09^ | 3.86 × 10^−07^ |
| Ubiquitin-specific protease activity | 72 | 377 | 4.1 | 2.0 | 1.78 × 10^−08^ | 3.98 × 10^−06^ |
| Ligase activity | 30 | 112 | 1.7 | 2.7 | 2 × 10^−07^ | 4.47 × 10^−05^ |
| Transporter activity | 94 | 576 | 5.3 | 1.7 | 3.68 × 10^−07^ | 8.25 × 10^−05^ |
| Hydrolase activity | 41 | 203 | 2.3 | 2.1 | 4.9 × 10^−06^ | 0.001097 |
| Cytoskeletal protein binding | 42 | 218 | 2.4 | 2.0 | 1.29 × 10^−05^ | 0.00288 |
| DNA binding | 96 | 654 | 5.4 | 1.5 | 2.78 × 10^−05^ | 0.006226 |
| Protein serine/threonine phosphatase activity | 14 | 44 | 0,8 | 3.3 | 4.55 × 10^−05^ | 0.010184 |
| Aminopeptidase activity | 11 | 29 | 0.6 | 3.9 | 4.79 × 10^−05^ | 0.010721 |
| Oxidoreductase activity | 32 | 161 | 1.8 | 2..0 | 7.13 × 10^−05^ | 0.015981 |
| Extracellular matrix structural constituent | 32 | 166 | 1.8 | 2.0 | 0.000131 | 0.02924 |

**Table S4.** An overview of 60 proteins showing detectable supernatant levels only for a minority of patients (≤10 patients corresponding to 25% of the patients) when AML cells were cultured alone (i.e. AML conditioned medium, referred to as AML-CM) but showing detectable levels for at least 30 patients (i.e. ≥75% of the patients) in MSC cultures prepared with the corresponding AML-CM plus fresh medium (1:1 ratio) (referred to as MSC/AML-CM). All the 60 proteins were released at high levels by the MSC when cultured alone. Yellow color marks proteins important for the extracellular matrix, green color cell surface proteins/adhesion molecules and blue color proteases/enzymes.

| **ABI3BP** | ***ABI family member 3 binding protein.* This is an extracellular matrix molecule involved in cell-matrix interaction (PMID 30923703).** | **Extracellular matrix** |
| --- | --- | --- |
| B4GALT1 | *Beta-1,4-galactosyltransferase 1.* This protein is a type II membrane-bound glycoproteins that transfers galactose in a beta1,4 linkage to similar acceptor sugars. Each of the seven beta4GalT has a distinct function in the biosynthesis of different glycoconjugates and saccharide structures. As type II membrane proteins, they have an N-terminal hydrophobic signal sequence that directs the protein to the Golgi apparatus and which then remains uncleaved to function as a transmembrane anchor. This gene is unique among the beta4GalT genes because it encodes an enzyme that participates both in glycoconjugate and lactose biosynthesis. For the first activity, the enzyme adds galactose to N-acetylglucosamine residues that are either monosaccharides or the nonreducing ends of glycoprotein carbohydrate chains. The second activity is restricted to lactating mammary tissues where the enzyme forms a heterodimer with alpha-lactalbumin to catalyze UDP-galactose + D-glucose <=> UDP + lactose. The two enzymatic forms result from alternate transcription initiation sites and post-translational processing. Two transcripts, which differ only at the 5' end, with approximate lengths of 4.1 kb and 3.9 kb encode the same protein. The longer transcript encodes the type II membrane-bound, trans-Golgi resident protein involved in glycoconjugate biosynthesis. The shorter transcript encodes a protein which is cleaved to form the soluble lactose synthase. | Protein glycosylation  Cell surface-Golgi  Lactose biosynthesis |
| BGN | *Biglycan.* This gene encodes a small leucine-rich proteoglycan. The encoded preproprotein is proteolytically processed to generate the mature protein, which plays a role in bone growth and collagen fibril assembly. The protein may also regulate inflammation and innate immunity. | Collagen  Bone growth |
| C1R | *Complement C1r.* This gene encodes a member of the peptidase S1 protein family. Mutations are associated with Ehlers-Danlos Syndrome. | Serine protease  Complement |
| CD248 | *CD248.* CD248/endosialin/TEM1 is a type 1 transmembrane glycoprotein found on the plasma membrane of activated mesenchymal cells. CD248 is expressed at high levels by malignant sarcoma cells, by the pericyte component of tumor vasculature and by mesenchymal cells in some fibrotic diseases (PMID 30847027). Loss-of-function studies in mice support the notion that CD248 promotes tumor growth (PMID 22206249). | Transmembrane glycoprotein  Tumor growth enhancement?  Angiogenesis? |
| CDH11 | *Cadherin 11.* This gene encodes a type II classical cadherin from the cadherin superfamily, a family of integral membrane proteins that mediate calcium-dependent cell-cell adhesion. Mature cadherin proteins are composed of a large N-terminal extracellular domain, a single membrane-spanning domain, and a small, highly conserved C-terminal cytoplasmic domain. Type II (atypical) cadherins are defined based on their lack of a HAV cell adhesion recognition sequence specific to type I cadherins. Expression of this particular cadherin in osteoblastic cell lines, and its upregulation during differentiation, suggests a specific function in bone development and maintenance. | Cell adhesion  Bone development |
| CDH13 | *Cadherin 12.* This gene encodes a member of the cadherin superfamily. The encoded protein is localized to the surface of the cell membrane and is anchored by a GPI moiety, rather than by a transmembrane domain. The protein lacks the cytoplasmic domain characteristic of other cadherins and is not thought to be a cell-cell adhesion glycoprotein. The gene is hypermethylated in many types of cancer. | Cell surface |
| CDH2 | *Cadherin 2.* This gene encodes a classical cadherin and member of the cadherin superfamily. Alternative splicing results in multiple transcript variants, at least one of which encodes a preproprotein is proteolytically processed to generate a calcium-dependent cell adhesion molecule and glycoprotein. This protein plays a role in formation of cartilage and bone. | Adhesion molecule |
| CFH | *Complement factor H.* This gene encodes a protein that has an essential role in the regulation of complement activation. Mutations in this gene have been associated with hemolytic-uremic syndrome (HUS) and chronic hypocomplementemic nephropathy. | Complement |
| COL10A1 | *Collagen type X alpha 1 chain.* This short chain collagen is expressed by hypertrophic chondrocytes during endochondral ossification. Unlike type VIII collagen, the other short chain collagen, type X collagen is a homotrimer. | Collagen |
| COL16A1 | *Collagen type XVI alpha 1 chain.* This is a member of the FACIT collagen family (fibril-associated collagens with interrupted helices). Members of this collagen family are found in association with fibril-forming collagens such as type I and II, and serve to maintain the integrity of the extracellular matrix. | Collagen |
| COL18A1 | *Collagen type XVIII alpha 1 chain.* This collagen is one of the multiplexins, extracellular matrix proteins that contain multiple triple-helix domains (collagenous domains) interrupted by non-collagenous domains. Proteolytic processing at several endogenous cleavage sites in the C-terminal domain results in production of endostatin, a potent antiangiogenic protein that is able to inhibit angiogenesis and tumor growth. | Collagen  Endostatin |
| COL4A1 | *Collagen type IV alpha 1 chain.* Type IV collagen proteins are integral components of basement membranes. It functions as part of a heterotrimer and interacts with other extracellular matrix components such as perlecans, proteoglycans, and laminins. In addition, proteolytic cleavage of the non-collagenous carboxy-terminal domain results in a biologically active fragment known as arresten, which has anti-angiogenic and tumor suppressor properties. | Collagen  Perlecan, laminin, proteoglycans  Arrestin |
| COL8A1 | *Collagen type VIII alpha 1 chain.* This gene encodes one of the two alpha chains of type VIII collagen. The protein is a short chain collagen and a major component of basement membranes. The type VIII collagen fibril can be either a homo- or a heterotrimer. | Collagen |
| CRIM1 | *Cysteine rich transmembrane BMP regulator 1.* The protein is regarded as a cancer-associated factor and interacts with various growth factors including TGFβs, BMPs, VEGFs and PDFGs (PMID 27044529, 25088037). | Cytokine modulation |
| CTGF | *Cellular communication network factor 2.* The protein is a mitogen that is also secreted by vascular endothelial cells. The encoded protein plays a role in chondrocyte proliferation/differentiation and cell adhesion. Certain polymorphisms in this gene have been linked with a higher incidence of systemic sclerosis. | Cell adhesion  Fibrosis |
| CTHRC1 | *Collagen triple helix repeat containing 1.* This protein is possibly involved in vascular remodeling. | Extracellular matrix |
| CTSK | *Cathepsin K.* This is a lysosomal cysteine proteinase involved in bone remodeling and resorption. The protein may contribute to tumor invasiveness and seems to be involved in the formation of extracellular matrix (PMID29781506). | Bone remodeling  Extracellular matrix? |
| CYR61 | *Cellular communication network factor 1.* This secreted protein is growth factor-inducible and promotes the adhesion of endothelial cells. The encoded protein interacts with several integrins and with heparan sulfate proteoglycan. This protein also plays a role in cell proliferation, differentiation, angiogenesis, apoptosis, and extracellular matrix formation. | Extracellular matrix  Cell adhesion  Cell proliferation |
| DAG1 | *Dystroglycan 1.* This protein is a component of dystrophin-glycoprotein complex that links the extracellular matrix and the cytoskeleton. The encoded preproprotein undergoes O- and N-glycosylation, and proteolytic processing to generate alpha and beta subunits. | Extracellular matrix  Cytoskeleton |
| DKK3 | *Dickkopf WNT signaling pathway inhibitor 3.* This member of the dickkopf family interacts with the Wnt signaling pathway and it may function as a tumor suppressor gene. | Wnt signaling |
| ECM1 | *Extracellular matrix protein 3.* This soluble protein is involved in endochondral bone formation, angiogenesis, and tumor biology. It also interacts with a variety of extracellular and structural proteins. | Extracellular matrix |
| ENPP1 | *Ectonucleotide pyrophosphatase/phosphodiesterase 1.* The encoded protein is a type II transmembrane glycoprotein comprising two identical disulfide-bonded subunits. This protein has broad specificity and cleaves a variety of substrates, including phosphodiester bonds of nucleotides and nucleotide sugars and pyrophosphate bonds of nucleotides and nucleotide sugars. This protein may function to hydrolyze nucleoside 5' triphosphates to their corresponding monophosphates and may also hydrolyze diadenosine polyphosphates. | Transmembrane glycoprotein |
| ENPP2 | *Ectonucleotide pyrophosphatase/phosphodiesterase 2.* The protein functions as both a phosphodiesterase, which cleaves phosphodiester bonds at the 5' end of oligonucleotides, and a phospholipase, which catalyzes production of lysophosphatidic acid in extracellular fluids. LPA evokes growth factor-like responses including stimulation of cell proliferation and chemotaxis. This gene product stimulates motility of tumor cells and has angiogenic properties, and its expression is upregulated in several carcinomas. The gene product is secreted and further processed to make the biologically active form. | Secreted enzyme  Growth factor  Angiogenic |
| FBLN1 | *Fibulin 1.* Fibulin 1 is a secreted glycoprotein that becomes incorporated into a fibrillar extracellular matrix. Calcium-binding is apparently required to mediate its binding to laminin and nidogen. It mediates platelet adhesion via binding fibrinogen. | Extracellular matrix |
| FBLN5 | Fibulin 5. The protein is a secreted, extracellular matrix protein containing an Arg-Gly-Asp (RGD) motif and calcium-binding EGF-like domains. It promotes adhesion of endothelial cells through interaction of integrins and the RGD motif. It may play a role in vascular development and remodeling. | Extracellular matrix  Vascular remodelling |
| FKBP10 | *FKBP prolyl isomerase 10.* The protein belongs to the FKBP-type peptidyl-prolyl cis/trans isomerase (PPIase) family. It localizes to the endoplasmic reticulum and acts as a molecular chaperone. | Endoplasmic reticulum  Chaperone |
| GAS6 | *Growth arrest specific 6.* This gamma-carboxyglutamic acid (Gla)-containing protein is thought to be involved in the stimulation of cell proliferation. This gene is frequently overexpressed in many cancers and has been implicated as an adverse prognostic marker. | Growth factor  Cancer prognosis |
| GOLM1 | *Golgi membrane protein 1.* The Golgi complex plays a key role in the sorting and modification of proteins exported from the endoplasmic reticulum. This protein is a type II Golgi transmembrane protein. It processes proteins synthesized in the rough endoplasmic reticulum and assists in the transport of protein cargo through the Golgi apparatus. | Golgi  Protein transport and secretion |
| GREM1 | *Remlin 1, DAN family BMP antagonist.* This BMP (bone morphogenic protein) antagonist family and a member of the CAN (cerberus and dan) subfamily of BMP antagonists. The antagonistic effect of this secreted glycosylated protein is likely due to its direct binding to BMP proteins. As an antagonist of BMP, this gene may play a role in organogenesis and tissue differentiation. | BMP antagonist |
| IGFBP4 | *Insulin like growth factor binding protein 1.* This insulin-like growth factor binding protein circulates in the plasma and binds both insulin-like growth factors (IGFs) I and II, prolonging their half-lives and altering their interaction with cell surface receptors. This protein is important in cell migration and metabolism. | IGF half-life and receptor binding |
| ISLR | *Immunoglobulin superfamily containing leucine rich repeat.*This 428 amino acid protein is possibley involved in binding of collagen or integrins to cell suraces (PMID 10512678, 15507277). | Extracellular matrix binding? |
| ITGBL1 | *Integrin subunit beta like 1.* This beta integrin-related protein that is a member of the EGF-like protein family. It contains integrin-like cysteine-rich repeats. | Integrin |
| LAMA4 | *Laminin subunit alpha 4.* Laminins are extracellular matrix proteins and the major noncollagenous constituent of basement membranes. They have been implicated in a wide variety of biological processes including cell adhesion, differentiation, migration, signaling and metastasis. Laminins are composed of 3 non identical chains: laminin alpha, beta and gamma (formerly A, B1, and B2, respectively). Each laminin chain is a multidomain protein encoded by a distinct gene. Several isoforms of each chain have been described. Different alpha, beta and gamma chain isomers combine to give rise to different heterotrimeric laminin isoforms. The biological functions of the different chains and trimer molecules are largely unknown. This gene encodes the alpha chain isoform laminin, alpha 4. | Extracellular matrix |
| LOX | *Lysyl oxidase.* This gene encodes a member of the lysyl oxidase family of proteins. Alternative splicing results in multiple transcript variants, at least one of which encodes a preproprotein that is proteolytically processed to generate a regulatory propeptide and the mature enzyme. The copper-dependent amine oxidase activity of this enzyme functions in the crosslinking of collagens and elastin, while the propeptide may play a role in tumor suppression. | Extracellular matrix  Tumor suppression? |
| LOXL1 | *Lysyl oxidase like 1.* This protein is a member of the lysyl oxidase family of proteins. The prototypic member of the family is essential to the biogenesis of connective tissue, encoding an extracellular copper-dependent amine oxidase that catalyzes the first step in the formation of crosslinks in collagen and elastin. The encoded preproprotein is proteolytically processed to generate the mature enzyme. The N-terminus is poorly conserved and may impart additional roles in developmental regulation, senescence, tumor suppression, cell growth control, and chemotaxis to each member of the family. | Extracellular matrix |
| LOXL2 |  | Extracellular matrix |
| MFAP2 | *Microfibril associated protein 2.* This protein is a major antigen of elastin-associated microfibrils. | Extracellular matrix |
| MMP13 | *Matrix metallopeptidase 13.* This is a member of the peptidase M10 family of matrix metalloproteinases (MMPs) that are involved in the breakdown of extracellular matrix in normal physiological processes as well as in disease processes, such as arthritis and metastasis. The encoded preproprotein is proteolytically processed to generate the mature protease. This protease cleaves type II collagen more efficiently than types I and III. | Extracellular matrix  Protease |
| MMP14 | *Matrix metallopeptidase 14.* Proteins of the matrix metalloproteinase (MMP) family are involved in the breakdown of extracellular matrix in normal physiological as well as in disease processes. The protein encoded by this gene is a member of the membrane-type MMP subfamily; these proteins contain a potential transmembrane domain suggesting that these proteins are expressed at the cell surface rather than secreted. This protein activates MMP2 protein, and this activity may be involved in tumor invasion. | Protease  Cell surface |
| MXRA8 | *Matrix remodeling associated 8.* The molecule is expressed by cancer-associated stromal cells and seems to be an entry receptor (PMID 25864925, 29769725). | Cell surface receptor |
| NBL1 | *NBL1, DAN family BMP antagonist.* This is a member of the CAN (Cerberus and DAN) family of proteins, which contain a domain resembling the CTCK (C-terminal cystine knot-like) motif found in a number of signaling molecules. These proteins are secreted, and act as BMP (bone morphogenetic protein) antagonists by binding to BMPs and preventing them from interacting with their receptors. | BMP antagonist |
| NRP2 | *Neuropilin 2.* This is a member of the neuropilin family of receptor proteins. This transmembrane protein binds to SEMA3C protein {sema domain, immunoglobulin domain (Ig), short basic domain, secreted, (semaphorin) 3C} and SEMA3F protein {sema domain, immunoglobulin domain (Ig), short basic domain, secreted, (semaphorin) 3F}, and interacts with vascular endothelial growth factor (VEGF). This protein may play a role in cardiovascular development and tumorigenesis. | VEGF  Angiogenesis? |
| OLFML2B | *Olfactormedin like 2B.* This gene encodes an olfactomedin domain-containing protein. Most olfactomedin domain-containing proteins are secreted glycoproteins. OLFML2B seems to participate in the regulation of multiple biological processes including cell growth, cell cycle regulation, apoptosis and cell communication through multiple signaling pathways including the M/G1 transition pathway, post-translational protein modification and DNA replication pre-initiation (PMID 31370831, 27821182). | Intracellular signaling |
| PAPPA | *Pappalysin 1.* This protein is a secreted metalloproteinase which cleaves insulin-like growth factor binding proteins (IGFBPs). Following IGFBP cleavage, insulin growth factors dissociate from IGFBPs and bind to IGF receptors, resulting in activation of the IGF pathway. The encoded protein plays a role in bone formation, inflammation and wound healing. | Metalloproteinase  IGF |
| PLOD2 | *Procollagen-lysine,2-oxoglutarate 5-dioxygenase 1.* This is a membrane-bound homodimeric protein localized to the cisternae of the endoplasmic reticulum. The enzyme catalyzes the hydroxylation of lysyl residues in collagen-like peptides. The resultant hydroxylysyl groups are attachment sites for carbohydrates in collagen and thus are critical for the stability of intermolecular crosslinks. | Lysyl hydroxylation  Collagen  Endoplasmatic reticulum |
| PLTP | *Phospholipid transfer protein.* The protein is one of at least two lipid transfer proteins found in human plasma. The encoded protein transfers phospholipids from triglyceride-rich lipoproteins to high density lipoprotein (HDL). In addition to regulating the size of HDL particles, this protein may be involved in cholesterol metabolism. | Lipid metabolism |
| PROCR | *Protein C receptor.* The protein is a receptor for activated protein C, a serine protease activated by and involved in the blood coagulation pathway. It an N-glycosylated type I membrane protein that enhances the activation of protein C. It has also been associated with cancer. | Serine protease |
| PRSS23 | *Serine protease 23.* This protein is a member of the trypsin family of serine proteases. | Serine protease |
| PTPRK | *Protein tyrosine phosphatase receptor type K.* The protein is a member of the protein tyrosine phosphatase (PTP) family. PTPs are signaling molecules that regulate a variety of cellular processes. This PTP possesses an extracellular region, a single transmembrane region, and two catalytic domains. It mediates homophilic intercellular interaction, possibly through the interaction with beta- and gamma-catenin at adherens junctions. Expression of this gene is stimulated by TGF-beta 1. | Tyrosine phosphatase |
| SDC1 | *Syndecan 1.* This is a transmembrane (type I) heparan sulfate proteoglycan and is a member of the syndecan proteoglycan family. The syndecans mediate cell binding, cell signaling, and cytoskeletal organization. The syndecan-1 protein functions as an integral membrane protein and participates in cell proliferation, cell migration and cell-matrix interactions via its receptor for extracellular matrix proteins. | Proteoglycan  Extracellular matrix  Migration |
| SMOC1 | *SPARC related modular calcium binding 1.* This is a multi-domain secreted protein. It is an ALK5 antagonist that tips TGF-β signalling towards ALK1 activation, thus promoting endothelial cell proliferation and angiogenesis (PMID 25750188). | Angiogenesis |
| SPON2 | *Spondin 2.* This is a proto-oncogene matrix protein that functions as a guanine nucleotide exchange factor through the activation of RhoGTPase. It is a prominent downstream signaling target of metastasis-associated in colon cancer 1 (MACC1) (PMID 27669439, 31691494, 28772159). | Intracellular signaling |
| SRPX2 | *Sushi repeat containing protein X-linked 2.* This secreted protein contains three sushi repeat motifs and may be involved in angiogenesis. | Angiogenesis? |
| SSC5D | *Scavenger receptor cysteine rich family member with 5 domains.* The scavenger receptor cysteine-rich (SRCR) family comprises a group of membrane-attached or secreted proteins that contain one or more modules/domains structurally similar to the membrane distal domain of type I macrophage scavenger receptor (PMID 27790215). | Cell surface receptor  Soluble form |
| STC2 | *Stannicalcin 2.* This gene encodes a secreted, homodimeric glycoprotein that may have autocrine or paracrine functions. It is phosphorylated by casein kinase 2 exclusively on its serine residues. The protein may play a role in the regulation of cell metabolism or cellular calcium/phosphate homeostasis. | Metabolism?  Calcium/phosphate homeostasis? |
| TAGLN | *Transgelin.* This gene encodes an actin-binding protein which belongs to the calponin family. It acts as a tumor suppressor, and knockout mice exhibit alterations in the distribution of the actin filament and changes in cytoskeletal organization. | Actin  Cytoskeleton |
| THY1 | *Thy-1 cell surface antigen.* This cell surface glycoprotein is involved in cell adhesion and cell communication in numerous cell types. It is a marker for hematopoietic stem cells and may function as a tumor suppressor. | Cell adhesion |
| TNC | *Tenascin C.* This extracellular matrix protein contains multiple EGF-like and fibronectin type-III domains. It is involved in cell migration. | Extracellular matrix |
| VASN | *Vasorin.* Vasorin is a transmembrane glycoprotein that interacts with the TGF-β and Notch1 pathways (PMID: **30564578).** | TGF/Wnt signaling |

**Table S5.** An overview of proteins with significantly increased or decreased levels in the supernatants of MSC cultures supplemented with AML conditioned medium (i.e. MSC/AML-CM) compared with the corresponding conditioned medium (i.e. AML-CM) (Log_2_ Fold change ≥1≤-2, *p*-value < 0.05). The table is sorted according to fold change (positive value: increased in MSC/AML-CM, negative value: decreased in MSC/AML-CM). These proteins included several extracellular matrix molecules and soluble adhesion molecules; these are marked with yellow in the table. *p*-value was determined by a paired *t*-test.

| **Protein Name** | **Gene Name** | **Detected in #MSCs** | **Detected in #AML-CM** | **Fold Change** | ***p*-value** |
| --- | --- | --- | --- | --- | --- |
| Collagen alpha-1(I) chain | COL1A1 | 7 | 40 | 9.79 | 5.318E-21 |
| Glia-derived nexin | SERPINE2 | 7 | 24 | 8.69 | 4.087E-10 |
| Periostin | POSTN | 7 | 36 | 8.22 | 3.647E-19 |
| Collagen alpha-2(VI) chain | COL6A2 | 7 | 39 | 8.13 | 2.947E-18 |
| Collagen alpha-3(VI) chain | COL6A3 | 7 | 40 | 8.09 | 1.077E-19 |
| Stanniocalcin-2 | STC2 | 7 | 7 | 7.93 | 4.070E-02 |
| Thrombospondin-2 | THBS2 | 7 | 25 | 7.78 | 2.617E-10 |
| SPARC | SPARC | 7 | 36 | 7.63 | 9.519E-16 |
| Collagen alpha-2(I) chain | COL1A2 | 7 | 40 | 7.53 | 2.791E-18 |
| Fibronectin | FN1 | 7 | 40 | 7.40 | 1.472E-17 |
| Fibrillin-1 | FBN1 | 7 | 34 | 7.25 | 4.084E-12 |
| Latent-transforming growth factor beta-binding protein 2 | LTBP2 | 7 | 20 | 7.20 | 2.535E-09 |
| Follistatin-related protein 1 | FSTL1 | 7 | 20 | 6.97 | 2.959E-10 |
| Lumican | LUM | 7 | 28 | 6.96 | 4.872E-09 |
| Collagen alpha-2(V) chain | COL5A2 | 7 | 24 | 6.60 | 7.309E-10 |
| Semaphorin-7A | SEMA7A | 7 | 21 | 6.57 | 6.671E-08 |
| 72 kDa type IV collagenase | MMP2 | 7 | 38 | 6.55 | 8.154E-18 |
| Collagen alpha-1(XII) chain | COL12A1 | 7 | 39 | 6.46 | 5.626E-16 |
| Collagen alpha-1(VI) chain | COL6A1 | 7 | 39 | 6.45 | 6.531E-15 |
| Collagen alpha-1(III) chain | COL3A1 | 7 | 38 | 6.43 | 2.206E-18 |
| Collagen alpha-1(V) chain | COL5A1 | 7 | 40 | 6.37 | 3.624E-17 |
| Basement membrane-specific heparan sulfate proteoglycan core protein | HSPG2 | 7 | 30 | 6.25 | 1.569E-12 |
| Transforming growth factor-beta-induced protein ig-h3 | TGFBI | 7 | 39 | 6.08 | 5.712E-18 |
| Decorin | DCN | 7 | 36 | 5.81 | 1.588E-09 |
| Insulin-like growth factor-binding protein 7 | IGFBP7 | 7 | 29 | 5.77 | 1.168E-09 |
| Serotransferrin | TF | 7 | 26 | 5.58 | 1.678E-10 |
| Connective tissue growth factor | CTGF | 7 | 7 | 5.48 | 3.154E-04 |
| Latent-transforming growth factor beta-binding protein 1 | LTBP1 | 7 | 19 | 5.45 | 1.030E-07 |
| Plasminogen activator inhibitor 1 | SERPINE1 | 7 | 39 | 5.10 | 3.895E-17 |
| Target of Nesh-SH3 | ABI3BP | 7 | 8 | 5.00 | 3.761E-02 |
| Hyaluronan and proteoglycan link protein 1 | HAPLN1 | 7 | 19 | 4.95 | 1.054E-07 |
| Fibulin-5 | FBLN5 | 7 | 4 | 4.48 | 1.083E-02 |
| Procollagen C-endopeptidase enhancer 1 | PCOLCE | 7 | 18 | 4.44 | 3.666E-05 |
| CD109 antigen | CD109 | 7 | 32 | 4.34 | 2.311E-11 |
| Complement C1s subcomponent | C1S | 7 | 14 | 4.32 | 5.470E-05 |
| Lysyl oxidase homolog 2 | LOXL2 | 7 | 9 | 4.31 | 3.820E-02 |
| Testican-1 | SPOCK1 | 7 | 15 | 4.27 | 4.360E-05 |
| Collagen alpha-1(XI) chain | COL11A1 | 7 | 22 | 4.26 | 1.266E-08 |
| Adipocyte enhancer-binding protein 1 | AEBP1 | 7 | 20 | 4.15 | 3.804E-06 |
| Inhibin beta A chain | INHBA | 7 | 12 | 4.01 | 6.321E-03 |
| Transgelin | TAGLN | 7 | 8 | 3.97 | 1.713E-03 |
| Metalloproteinase inhibitor 1 | TIMP1 | 7 | 33 | 3.95 | 1.005E-09 |
| Olfactomedin-like protein 3 | OLFML3 | 7 | 11 | 3.95 | 3.948E-03 |
| Serine protease HTRA1 | HTRA1 | 7 | 15 | 3.91 | 2.351E-03 |
| Inactive tyrosine-protein kinase 7 | PTK7 | 7 | 24 | 3.87 | 1.304E-08 |
| Peroxidasin homolog | PXDN | 7 | 12 | 3.78 | 4.232E-03 |
| Insulin-like growth factor-binding protein 6 | IGFBP6 | 7 | 13 | 3.77 | 4.585E-05 |
| Interstitial collagenase | MMP1 | 7 | 14 | 3.69 | 1.297E-06 |
| Sulfhydryl oxidase 1 | QSOX1 | 7 | 23 | 3.66 | 2.092E-06 |
| Ectonucleotide pyrophosphatase/phosphodiesterase family member 2 | ENPP2 | 7 | 10 | 3.53 | 4.734E-02 |
| Pigment epithelium-derived factor | SERPINF1 | 7 | 34 | 3.40 | 2.751E-11 |
| Metalloproteinase inhibitor 2 | TIMP2 | 7 | 27 | 3.40 | 2.113E-09 |
| Collagen alpha-1(VII) chain | COL7A1 | 7 | 19 | 3.40 | 9.749E-07 |
| Clusterin;Clusterin beta chain;Clusterin alpha chain | CLU | 7 | 24 | 3.36 | 1.390E-07 |
| Amyloid beta A4 protein | APP | 7 | 15 | 3.31 | 1.139E-04 |
| Procollagen-lysine,2-oxoglutarate 5-dioxygenase 1 | PLOD1 | 7 | 14 | 3.27 | 3.683E-04 |
| EMILIN-1 | EMILIN1 | 7 | 30 | 3.24 | 3.248E-08 |
| Urokinase-type plasminogen activato | PLAU | 7 | 13 | 3.19 | 2.053E-05 |
| Sushi repeat-containing protein SRPX | SRPX | 7 | 16 | 3.15 | 3.928E-04 |
| Tyrosine-protein kinase receptor UFO | AXL | 7 | 16 | 3.10 | 1.932E-04 |
| Gremlin-1 | GREM1 | 7 | 7 | 3.07 | 8.710E-03 |
| Pre-mRNA-splicing factor 38A | PRPF38A | 1 | 21 | 3.06 | 4.034E-03 |
| Thrombospondin-1 | THBS1 | 7 | 40 | 3.04 | 4.580E-10 |
| Collagen triple helix repeat-containing protein 1 | CTHRC1 | 7 | 8 | 3.02 | 2.608E-04 |
| Beta-glucuronidase | GUSB | 1 | 13 | 2.97 | 1.098E-03 |
| Glypican-1;Secreted glypican-1 | GPC1 | 7 | 16 | 2.95 | 3.956E-04 |
| Versican core protein | VCAN | 7 | 26 | 2.95 | 2.955E-06 |
| Prolow-density lipoprotein receptor-related protein 1 | LRP1 | 7 | 22 | 2.95 | 2.507E-06 |
| Aminopeptidase N | ANPEP | 7 | 34 | 2.91 | 1.228E-11 |
| Metallothionein-2;Metallothionein-1X;Metallothionein-1G | MT2A | 6 | 33 | 2.86 | 1.529E-05 |
| Dynein heavy chain 14, axonemal | DNAH14 | 3 | 19 | 2.84 | 9.756E-04 |
| C-type lectin domain family 11 member A | CLEC11A | 7 | 32 | 2.83 | 2.688E-10 |
| Chondroitin sulfate proteoglycan 4 | CSPG4 | 7 | 19 | 2.83 | 4.305E-05 |
| Endothelial protein C receptor | PROCR | 7 | 10 | 2.77 | 8.700E-04 |
| Nidogen-1 | NID1 | 7 | 27 | 2.76 | 8.619E-07 |
| Reticulocalbin-3 | RCN3 | 7 | 19 | 2.72 | 9.864E-05 |
| Tricarboxylate transport protein, mitochondrial | SLC25A1 | 5 | 6 | 2.62 | 2.424E-02 |
| Calumenin | CALU | 7 | 36 | 2.54 | 2.739E-09 |
| Serpin H1 | SERPINH1 | 7 | 14 | 2.49 | 5.610E-03 |
| Fibulin-1 | FBLN1 | 7 | 13 | 2.47 | 3.453E-06 |
| C-type mannose receptor 2 | MRC2 | 7 | 14 | 2.41 | 7.600E-03 |
| Pentraxin-related protein PTX3 | PTX3 | 7 | 15 | 2.40 | 2.648E-02 |
| Insulin | INS | 5 | 14 | 2.31 | 3.968E-02 |
| Xylosyltransferase 1 | XYLT1 | 7 | 17 | 2.30 | 2.013E-04 |
| Ubiquitin carboxyl-terminal hydrolase isozyme L1 | UCHL1 | 7 | 16 | 2.29 | 3.549E-03 |
| Nucleobindin-1 | NUCB1 | 7 | 37 | 2.25 | 1.412E-08 |
| Extracellular matrix protein 1 | ECM1 | 7 | 10 | 2.25 | 3.669E-02 |
| EGF-containing fibulin-like extracellular matrix protein 2 | EFEMP2 | 7 | 25 | 2.24 | 1.689E-07 |
| Periostin | POSTN | 7 | 15 | 2.18 | 3.831E-02 |
| Neuropilin-1 | NRP1 | 7 | 14 | 2.11 | 1.554E-02 |
| Cartilage oligomeric matrix protein | COMP | 7 | 31 | 2.09 | 4.122E-06 |
| 5-nucleotidase | NT5E | 7 | 11 | 2.08 | 4.562E-04 |
| Caldesmon | CALD1 | 7 | 26 | 2.06 | 1.191E-06 |
| 45 kDa calcium-binding protein | SDF4 | 7 | 25 | 2.04 | 9.265E-06 |
| Fibronectin | FN1 | 7 | 20 | 2.02 | 2.172E-05 |
| Cathepsin B | CTSB | 7 | 37 | 2.00 | 2.998E-06 |
| Calsyntenin-1;Soluble Alc-alpha;CTF1-alpha | CLSTN1 | 7 | 31 | 1.97 | 5.070E-08 |
| Trans-Golgi network integral membrane protein 2 | TGOLN2 | 7 | 19 | 1.92 | 7.080E-05 |
| Superoxide dismutase [Mn], mitochondrial | SOD2 | 3 | 26 | 1.83 | 4.628E-08 |
| Spondin-2 | SPON2 | 5 | 8 | 1.75 | 3.553E-02 |
| Keratin, type II cytoskeletal 2 epidermal | KRT2 | 6 | 36 | 1.73 | 1.213E-02 |
| Soluble scavenger receptor cysteine-rich domain-containing protein SSC5D | SSC5D | 7 | 5 | 1.70 | 3.472E-02 |
| CD166 antigen | ALCAM | 7 | 26 | 1.70 | 3.230E-08 |
| Insulin-like growth factor-binding protein 3 | IGFBP3 | 7 | 25 | 1.67 | 1.190E-02 |
| CD81 antigen | CD81 | 7 | 17 | 1.65 | 6.261E-05 |
| Polypeptide N-acetylgalactosaminyltransferase 2 | GALNT2 | 6 | 18 | 1.60 | 7.735E-04 |
| Major prion protein | PRNP | 6 | 9 | 1.57 | 2.337E-02 |
| Plexin-B2 | PLXNB2 | 7 | 23 | 1.55 | 4.887E-07 |
| Thioredoxin reductase 1, cytoplasmic | TXNRD1 | 6 | 25 | 1.52 | 4.142E-05 |
| Disintegrin and metalloproteinase domain-containing protein 9 | ADAM9 | 7 | 14 | 1.51 | 3.207E-03 |
| Plexin domain-containing protein 2 | PLXDC2 | 7 | 25 | 1.49 | 6.350E-10 |
| Procollagen-lysine,2-oxoglutarate 5-dioxygenase 3 | PLOD3 | 6 | 22 | 1.49 | 4.720E-03 |
| Coiled-coil domain-containing protein 80 | CCDC80 | 7 | 27 | 1.45 | 1.140E-05 |
| Cystatin-C | CST3 | 7 | 34 | 1.43 | 1.801E-07 |
| CD82 antigen | CD82 | 0 | 12 | 1.43 | 4.084E-02 |
| Aspartate aminotransferase, mitochondrial | GOT2 | 7 | 36 | 1.41 | 5.890E-06 |
| Ganglioside GM2 activator;Ganglioside GM2 activator isoform short | GM2A | 4 | 24 | 1.41 | 8.268E-05 |
| Leucine-rich PPR motif-containing protein, mitochondrial | LRPPRC | 2 | 26 | 1.41 | 3.736E-02 |
| Protein S100-A6 | S100A6 | 6 | 33 | 1.40 | 2.171E-05 |
| CD59 glycoprotein | CD59 | 5 | 13 | 1.40 | 5.289E-03 |
| Myristoylated alanine-rich C-kinase substrate | MARCKS | 7 | 31 | 1.37 | 4.697E-06 |
| Glutathione reductase, mitochondrial | GSR | 4 | 34 | 1.37 | 7.568E-06 |
| EGF-containing fibulin-like extracellular matrix protein 1 | EFEMP1 | 4 | 11 | 1.34 | 6.576E-04 |
| Brain acid soluble protein 1 | BASP1 | 7 | 38 | 1.32 | 2.903E-06 |
| 4F2 cell-surface antigen heavy chain | SLC3A2 | 7 | 26 | 1.32 | 1.757E-07 |
| Zinc finger protein 638 | ZNF638 | 1 | 21 | 1.30 | 7.278E-04 |
| Disintegrin and metalloproteinase domain-containing protein 17 | ADAM17 | 0 | 6 | 1.26 | 6.966E-03 |
| Granulins | GRN | 6 | 34 | 1.25 | 4.053E-07 |
| Putative trypsin-6 | PRSS3P2 | 6 | 10 | 1.23 | 3.853E-03 |
| Src substrate cortactin | CTTN | 6 | 7 | 1.22 | 9.519E-03 |
| Non-specific lipid-transfer protein | SCP2 | 0 | 26 | 1.22 | 1.407E-04 |
| Protein S100-B | S100B | 0 | 13 | 1.21 | 2.239E-02 |
| Growth-regulated alpha protein | CXCL1 | 1 | 10 | 1.20 | 1.677E-02 |
| Proteasome subunit beta type-5 | PSMB5 | 7 | 39 | 1.16 | 3.554E-05 |
| Glutathione synthetase | GSS | 6 | 36 | 1.14 | 4.385E-10 |
| Rho-associated protein kinase 1 | ROCK1 | 4 | 37 | 1.11 | 1.099E-02 |
| Transforming growth factor beta-1 | TGFB1 | 7 | 11 | 1.07 | 5.854E-03 |
| Disintegrin and metalloproteinase domain-containing protein 10 | ADAM10 | 6 | 25 | 1.07 | 3.169E-04 |
| Neutral amino acid transporter B(0) | SLC1A5 | 5 | 19 | 1.06 | 1.929E-04 |
| Serglycin | SRGN | 7 | 39 | 1.06 | 5.771E-08 |
| Copine-3 | CPNE3 | 0 | 15 | 1.06 | 1.013E-02 |
| Laminin subunit gamma-1 | LAMC1 | 7 | 32 | 1.04 | 2.139E-03 |
| N(G),N(G)-dimethylarginine dimethylaminohydrolase 1 | DDAH1 | 6 | 14 | 1.02 | 9.337E-04 |
| TBC1 domain family member 5 | TBC1D5 | 7 | 21 | 1.02 | 2.941E-02 |
| Lymphoid-restricted membrane protein | LRMP | 0 | 17 | 1.02 | 5.459E-03 |
| Integral membrane protein 2B | ITM2B | 7 | 28 | 1.01 | 8.730E-05 |
| 14-3-3 protein sigma | SFN | 2 | 28 | -2.02 | 7.174E-03 |
| Septin-2 | SEPT2 | 2 | 40 | -2.03 | 5.065E-09 |
| Kynureninase | KYNU | 0 | 22 | -2.16 | 1.728E-06 |
| Hemoglobin subunit alpha | HBA1 | 7 | 40 | -2.18 | 1.743E-12 |
| 60S ribosomal protein L3 | RPL3 | 6 | 36 | -2.19 | 5.788E-06 |
| Stress-70 protein, mitochondrial | HSPA9 | 5 | 37 | -2.20 | 3.192E-07 |
| Citrate synthase, mitochondrial | CS | 0 | 33 | -2.25 | 1.008E-05 |
| Glutathione peroxidase 1 | GPX1 | 0 | 35 | -2.28 | 1.871E-06 |
| Histone H2A type 2-C | HIST2H2AC | 1 | 35 | -2.28 | 5.087E-03 |
| ATP synthase subunit beta, mitochondrial | ATP5B | 5 | 40 | -2.28 | 7.966E-14 |
| 60 kDa heat shock protein, mitochondrial | HSPD1 | 5 | 40 | -2.38 | 7.807E-12 |
| DNA-dependent protein kinase catalytic subunit | PRKDC | 3 | 38 | -2.46 | 2.365E-03 |
| Hemoglobin subunit beta | HBB | 6 | 40 | -2.48 | 5.981E-12 |
| 60S ribosomal protein L15 | RPL15 | 4 | 33 | -2.48 | 1.869E-07 |
| Prohibitin-2 | PHB2 | 0 | 30 | -2.49 | 4.672E-03 |
| Mycophenolic acid acyl-glucuronide esterase, mitochondrial | ABHD10 | 0 | 27 | -2.57 | 1.532E-02 |
| Enoyl-CoA hydratase, mitochondrial | ECHS1 | 0 | 31 | -2.60 | 1.292E-03 |
| Methionine adenosyltransferase 2 subunit beta | MAT2B | 0 | 25 | -2.62 | 6.872E-03 |
| Elongation factor Tu, mitochondrial | TUFM | 2 | 36 | -2.70 | 3.242E-07 |
| Hemoglobin subunit delta | HBD | 0 | 35 | -2.76 | 1.309E-08 |
| 3-ketoacyl-CoA thiolase, mitochondrial | ACAA2 | 4 | 32 | -2.82 | 8.204E-08 |
| Aconitate hydratase, mitochondrial | ACO2 | 4 | 34 | -2.90 | 1.846E-05 |
| Aconitate hydratase, mitochondrial | ACAT1 | 0 | 26 | -3.13 | 9.521E-03 |
| 3-hydroxyacyl-CoA dehydrogenase type-2 | HSD17B10 | 0 | 29 | -3.24 | 5.479E-03 |
| Electron transfer flavoprotein subunit alpha, mitochondrial | ETFA | 3 | 32 | -3.43 | 1.485E-06 |
| Isocitrate dehydrogenase [NADP], mitochondrial | IDH2 | 0 | 36 | -3.84 | 2.803E-08 |

**Table S6.** Overview of the 1043 proteins belonging to the GO term Exosome, detected in the supernatants after in vitro culture of primary human AML cells. Cells from 40 AML patients were cultured in serum-free medium for 48 hours before supernatants were harvested and analyzed. The proteins are listed according to their gene name, and 82 proteins/genes marked with yellow belong to the top 100 list of exosomal proteins (<http://exocarta.org/exosome_markers_new>).

| **40 Patients** | **YWHAH** | **P4HB** | **ANXA2** | **PSMD2** | **PSMB4** | **PTPN6** |
| --- | --- | --- | --- | --- | --- | --- |
| HBB | HSPA8 | PSME1 | THBS1 | GLO1 | SERBP1 | CORO1A |
| HSPD1 | AHNAK | MYH9 | FN1 | VPS35 | S100A11 | CCT8 |
| ATP5B | DDB1 | RPL4 | COL1A2 | CAPZB | THRAP3 | CAND1 |
| HBA1 | ACTG1 | TXNDC5 | COL6A3 | H2AFY | CD44 | CLTC |
| SEPT2 | MYL6 | HSP90AA1 | PPIB | APRT | PSMA4 | HNRNPR |
| RBMX | RPSA | GSN | HPRT1 | TUBB4B | CAT | RAB14 |
| PGK1 | GSTP1 | MSN | RPS5 | YWHAQ | TPI1 | HIST1H2AC |
| ENO1 | DPYSL2 | HSP90AB1 | **39 patients** | ACTC1 | PSMB1 | PTPRC |
| PPIA | NCL | YWHAE | ATP5A1 | UBE2N | GPI | PFAS |
| HIST1H2AJ | HSP90B1 | PARK7 | HNRNPC | SUB1 | RNASET2 | EIF4A1 |
| GANAB | ACLY | ARHGDIB | H2AFV/Z | YWHAG | PGM2 | RPL10A |
| RNH1 | ILF3 | DBI | TALDO1 | CAPZA1 | PRDX1 | PAFAH1B1 |
| PFN1 | PDIA6 | PNP | RPS3A | DBNL | TPM4 | RPS9 |
| HNRNPA1 | EEF2 | LCP1 | HIST1H2BC | MVP | NUCB2 | PCMT1 |
| RAN | STMN1 | VIM | RPL5 | RAB1B | RPS27A | MYL12A |
| TAGLN2 | YWHAZ | NME2 | RPLP2 | STX7 | TXNRD1 | EIF6 |
| PGD | YWHAB | UGP2 | GAPDH | AKR1A1 | SOD1 | CAPZA2 |
| CLIC1 | RPS20 | TXN | PA2G4 | AK2 | PSMB5 | PSME2 |
| CCT7 | TPM3 | COTL1 | PPA1 | PSMB8 | SERPINE1 | PABPN1 |
| CFL1 | EEF1A1 | ARHGDIA | SUMO2 | PTBP1 | TGFBI | FAM49B |
| AHCY | HNRNPK | YBX1 | ACTB | IQGAP1 | COL6A1 | TSTA3 |
| CCT2 | ANP32B | TWF2 | RPS18 | ARPC2 | COL12A1 | EIF2S3 |
| RPL12 | PKM | LDHB | TUBB | MAN2B1 | COL6A2 | HIST1H3A |
| ALDOA | SH3BGRL3 | CALR | CCT3 | GLOD4 | **38 patients** | SNRPD2 |
| RPL11 | TLN1 | PSMA1 | TARS | ANXA1 | LAP3 | FASN |
| GNB1 | LTA4H | DPP3 | H2AFX | FABP5 | SND1 | UBE2V2 |
| ARPC4 | HSPA5 | PSMA5 | HNRNPD | FLNB | ALDH9A1 | PYGL |
| HNRNPL | SYNCRIP | FLNA | PPP2R4 | PRDX2 | IDH1 | SERPINB1 |
| PGAM1 | PDIA3 | SPTAN1 | PPP2R1A | PABPC1 | PAICS | PGLS |
| RPLP0/6 | FH | PSMA7 | CACYBP | CAPG | ACTR2 | NEDD8 |
| UBA1 | RPS3 | PSMA3 | WARS | SPTBN1 | RUVBL1 | CA1 |
| PEBP1 | TKT | MDH1 | MTAP | CANX | 39326 | VAMP8 |
| MDH2 | GDI2 | HIST1H4A | WDR1 | PSMA6 | RAB2A | PSMB9 |
| CCT5 | CDC37 | ACTN4 | GNB2 | VASP | TCP1 | SH3BGRL |
| HSPA4 | PRDX6 | VCL | ARPC1B | EEF1G | KPNB1 | GNPDA1 |
| HNRNPM | NUMA1 | LGALS1 | FSCN1 | FKBP4 | HSPE1 | LSP1 |
| CAP1 | GSTO1 | LDHA | CNDP2 | PSMB6 | ATP6V1A | PSMB3 |
| VCP | EZR | PLEC | RAB11B | PSMB2 | RPL23A | MPO |
| HNRNPA2B1 | ACTR3 | ACTN1 | HSPA1B/A | HDGF | RPL28 | UBE2V1 |
| RPS5 | HPRT1 | PPIB | PCBP1 | PSMA2 | RUVBL2 | PEPD |

| CTSZ | NUCB1 | UBE2D2 | **34 patients** | **33 patients** | ATP6V1F | RAB6A |
| --- | --- | --- | --- | --- | --- | --- |
| BASP1 | B2M | CFD | GRHPR | CS | SMS | PSMC6 |
| **37 patients** | PSAP | NPC2 | AKR1B1 | RPS2 | PSMD7 | PRCP |
| HSPA9 | CTSB | NME1 | CTSC | ITGB2 | H3F3A | LAMTOR1 |
| HSPH1 | **36 patients** | GSS | RAP1B | GLUL | SERPINA1 | RARS |
| RPS4X | IDH2 | GOT2 | NAPRT | QDPR | GLRX | GCA |
| DDAH2 | TUFM | KRT2 | NUTF2 | PHPT1 | PCBD1 | DDX19B |
| HINT1 | RPL3 | **35 patients** | EIF3B | RPL30 | OXSR1 | CAPNS1 |
| PPP1CA | DYNC1H1 | HIST2H2AC | HIST2H2BE | AKR1C3 | PRTN3 | MARCKSL1 |
| RHOA | ATIC | ESD | CTSG | RPS16 | STAM | MMP9 |
| RNPEP | G6PD | PGM1 | MIF | DAK | CSTB | IGFBP7 |
| RAB5C | ENO1 | RPS13 | ERAP1 | STK26 | MARCKS | **28 patients** |
| CCT6A | ARF3 | XPNPEP1 | EIF3H | ATP1A1 | COMP | SFN |
| SLC9A3R1 | TXNDC17 | UGGT1 | STK10 | TPP1 | SIRPA | WDR61 |
| COPA | PCBP2 | PFKL | SERPINB6 | CUTA | **30 patients** | CSNK2B |
| NANS | NAPA | MTHFD1 | RPS15A | ICAM3 | SERPINB9 | RPL35A |
| PDCD6IP | ECH1 | RPS8 | ACTR1A | RSU1 | PHB | CHMP4B |
| NPEPPS | GNB2L1 | DDT;DDTL | PYGB | IGHG3 | ACO1 | QPRT |
| ATP6V1B2 | RPS14 | GNAI2 | SEC13 | S100A6 | SLC25A3 | MNDA |
| APEH | RAC2 | PSMD3 | BLVRB | TIMP1 | S100P | STXBP2 |
| PRDX5 | FERMT3 | CDC42 | VAT1 | **32 patients** | NDRG1 | GARS |
| NUDT5 | PPP2CA | CCT4 | WAS | NIT2 | GART | MOB1A |
| RPS19 | ADH5 | ADSS | TNPO1 | CRYZ | MYH14 | CD14 |
| RAB7A | SRSF7 | CSE1L | RPE | CORO1B | FBP1 | LAMP2 |
| IQGAP2 | DCTN2 | NARS | SARS | CBR1 | C11orf54 | DSTN |
| ARPC5 | SRI | ATP6V1E1 | VTA1 | IMPDH2 | SERPINB8 | HSPB1 |
| HSPA6 | LAMTOR3 | EIF3I | UFC1 | AKR7A2 | AARS | ARF4 |
| NAGK | CAP1 | CMPK1 | ACTBL2 | HEBP1 | IARS | CLIC4 |
| GBE1 | PAFAH1B2 | PSMC5 | FMNL1 | RPL22 | PACSIN2 | RP2 |
| GRB2 | CTSD | DDX3X/Y | AP1M1 | BROX | ELANE | DES |
| ARPC3 | ALYREF | ABHD14B | ERP44 | CAD | TUBA4A | ITM2B |
| S100A4 | OTUB1 | ST13 | HYOU1 | RPL31 | PRKAR2A | LUM |
| NAMPT | FKBP5 | CAPN2 | UBE2I | GNG5 | ACP1 | DNPH1 |
| PDCD5 | PPP1R7 | ANXA6 | ALDOC | SDCBP | LAMP1 | **27 patients** |
| SUMO3 | USP14 | DARS | RDX | HUWE1 | APOE | HLA-DRA |
| DUT | CAPN1 | CAB39 | EHD1 | LAMC1 | TFRC | MPI |
| ELAVL1 | DCPS | DPP7 | CSRP1 | **31 patients** | APLP2 | PFKP |
| RPS11 | ATP6V1G1 | HLA-C | LMAN2 | ACAT2 | ITGB1 | CSK |
| ALAD | RPS28 | BAX | GGH | PCNA | EMILIN1 | SPN |
| EIF2S1 | GNS | HEXB | HEXA | PPP1CB | HSPG2 | NID2 |
| EEA1 | LYZ | ARHGAP1 | GRN | RPL14 | **29 patients** | FAM129A |
| TPT1 | AHSA1 | FAH | GSR | RHOG | GSTK1 | LYN |
| FBL | GGCT | HDHD2 | CST3 | SORD | EIF3E | PPT1 |
| SRRT | HEBP2 | CA2 | ANPEP | PECAM1 | MYO1G | CTSA |
| RPS26 | VPS4B | GOT1 | SERPINF1 | ASAH1 | PDXK | PSMD12 |
| C3 | BLMH | HIST2H3A | OLA1 | RPL26 | RPL23 | IGFBP2 |

| RNASE2 | A2M | DEFA3 | RPS25 | NCBP1 | PSAT1 | EFEMP1 |
| --- | --- | --- | --- | --- | --- | --- |
| S100A8 | GLIPR2 | ITIH1 | ACTR1B | CD37 | IGFALS | NT5E |
| MYOF | CREG1 | DNHD1 | SLC1A5 | RBP4 | SPAG9 | MOGS |
| BSG | MSRA | SQSTM1 | CARHSP1 | PLBD2 | DDAH1 | **10 patients** |
| NID1 | H2AFY2 | IL1RN | CD9 | F11R | ADAM9 | ITGB5 |
| **26 patients** | TOM1 | APOC3 | CSPG4 | ATRN | PLOD1 | SSR4 |
| ACAT1 | ATP6V1C1 | PRRC2A | EXOSC2 | FAM3C | **13 patients** | HLA-E |
| FUCA1 | FGA | SLK | EXOSC6 | CD81 | ACY3 | HLA-DRB1 |
| ITGAL | SDC4 | HP | FGL2 | **16 patients** | CD59 | PLA2G15 |
| CPNE1 | IGHG2 | PLOD3 | GP1BA | ACSL4 | FBLN1 | TTC38 |
| ITGAM | PITPNA | **21 patients** | ATP6V1D | GPC1 | CHMP2A | SPEN |
| NCKAP1L | CRK | S100A10 | GPX4 | AXL | GUSB | OSCAR |
| ABI1 | GM2A | ORM1 | HNRNPD | CTSH | PLAU | FBLN1 |
| DNM2 | CLU | AZU1 | LGMN | ITGA4 | EHD4 | FCER2 |
| IST1 | **23 patients** | CMBL | LY75 | DNAJA1 | FGG | SERPING1 |
| SAFB2 | ENO2 | PDCD10 | MPST | EPB41L2 | NQO2 | ECM1 |
| CD2AP | ALDH1A1 | TRHDE | PDCD6 | EXOSC4 | SCAMP3 | PROCR |
| DNAJB1 | ARL8B | PLAA | PRDX4 | PTGDS | VPS28 | LAMA4 |
| LGALS3 | ASNA1 | EXOSC9 | GSTM2 | LYPLA2 | **12 patients** | BGN |
| ANXA5 | PDDC1 | NAGA | SRPR | PRKACA | AASDHPPT | **9 patients** |
| SLC3A2 | CD74 | DNASE2 | CPVL | RAB27B | ARHGEF18 | C2 |
| ALCAM | CD97 | IGF2R | VWF | TMED9 | ATP6V1H | PRNP |
| SOD2 | LTF | **20 patients** | ZC3H14 | **15 patients** | BPGM | CP |
| TF | RPL24 | RAB3D | **18 patients** | LMAN1 | BPI | GNG2 |
| **25 patients** | VCAM1 | SHMT2 | ACY1 | CYFIP2 | CD82 | HNRNPR |
| MAT2B | IDE | ARSB | FTH1 | CFB | CD34 | LILRB4 |
| TMEM109 | TPM3 | FLOT1 | SPP1 | GGACT | PXDN | PYGM |
| PHGDH | RAP1GDS1 | RAB1A | PCOLCE | RALA | MAN1A1 | TSG101 |
| SLC4A1 | RAB8A | VAMP7 | DNAJA2 | EXOSC3 | GBA | TUBB6 |
| CUL3 | SKIV2L2 | FGB | DNAJC7 | ICAM1 | IGHM | VPS50 |
| TOLLIP | HSP90AB2P | LYPLA1 | FAM49B | CPNE3 | LRG1 | **8 patients** |
| GAA | UBE2G1 | ARSA | HNMT | APP | MYH10 | SLC2A1 |
| RAB10 | S100A9 | WASF2 | LUZP1 | HTRA1 | RAC1 | HBS1L |
| PRKACB | CTSL | EXOSC8 | PABPC4 | **14 patients** | STAU1 | BDH2 |
| CHMP5 | JUP | GMDS | PRKCB | ZCCHC8 | LAMB1 | RENBP |
| ASL | LGALS3BP | DCXR | RAB5B | EIF4E | **11 patients** | SCRN2 |
| IGHA1 | PLXNB2 | ATP6AP2 | PTER | COPS8 | DYSF | CYFIP1 |
| ADAM10 | QSOX1 | FAM129B | RAB11A | PLCG2 | RAB8B | PTGR1 |
| TXNRD1 | **22 patients** | FTL | GALM | IGJ | TPM3 | BST1 |
| SDF4 | TUBB8 | ATP6AP1 | TOMM70A | GALK1 | HPX | GNB4 |
| EFEMP2 | RPL37A | C4A | COL4A2 | GALNS | BST2 | GP5 |
| **24 patients** | NT5C | FN1 | VPS13C | ACOT7 | STOM | CHI3L1 |
| VDAC1 | APPL1 | AEBP1 | ZC3H18 | APOB | ITGB3 | TPM4 |
| BLVRA | RAB27A | FSTL1 | **17 patients** | PTPRJ | TGM2 | SPON2 |
| RPL27 | B4GAT1 | LTBP2 | SELENBP1 | AGRN | FABP4 | MXRA8 |
| IGLC6 | TUBA1A | **19 patients** | SSBP1 | IGKC | QPCT | **7 patients** |

| SPINT1 | F2 | GP6 |
| --- | --- | --- |
| ALDH2 | COL18A1 | MGAT1 |
| STK24 | CFH | ARFGEF2 |
| PRKCD | CDH11 | DNAJC3 |
| RAB21 | GC | PPIC |
| SNX18 | C8B | PAM |
| CEACAM8/1 | LSP1 | COL6A2 |
| SCPEP1 | GMPPA | FN1 |
| C1R | PADI2 | MYL12B |
| CDH13 | **4 patients** | AGT |
| **6 patients** | ARPC1A | CALML3 |
| BTD | DAG1 | STX4 |
| AGAP2 | FBLN5 | DSP |
| ANGPT1 | DCD | HMCN1 |
| ARF1 | DSC2 | EXT2 |
| C9 | EPB41L2 | **1 patient** |
| CD300A | HRG | SDC1 |
| CHID1 | HSPA13 | AHSG |
| SLC25A1 | IL1B | PLOD2 |
| FCGR3B | LAMB2 | AMY2B |
| FGR | MAN2B2 | AOC1 |
| GNPTG | MGAM | APOA2 |
| H2AFY | IGKV3D-20 | ARRDC1 |
| HGS | RETN | AZGP1 |
| HRSP12 | TUBB4A | CD48 |
| ICOSLG | **4 patients** | DNAJC13 |
| LGALS7 | AKR1C1 | DPYSL2 |
| GAS6 | MFGE8 | EFNB1 |
| SCAMP2 | B4GALT1 | FABP3 |
| **5 patients** | CNP | GSTM3 |
| ANXA3 | CST6 | HNRNPC |
| ISLR | EXOSC5 | LCN2 |
| UGP2 | ITIH4 | FLRT2 |
| PTPRG | NDRG2 | OLFM4 |
| RFC1 | NPR3 | PROS1 |
| CANT1 | OGN | PLD3 |
| GNAQ | PKLR | EDIL3 |
| APOA1 | POTEI | LTBP3 |
| HLA-DRB5 | **2 patients** | TTR |
| ARL3 | CPE | GNG12 |
| NAGLU | ASS1 | WNT5B |
| GNAS | PRSS23 |  |
| SHMT1 | COL6A3 |  |
| CD248 | CLEC3B |  |
| VASN | IGHG4 |  |
| LEPRE1 | SERPINA3 |  |

**Table S7.** Effects of AML conditioned medium on the constitutive protein release by MSC. The table lists all proteins that were (i) constitutively released by ≥4 MSC and ≤2 AML cell populations/patients cultured alone; and (ii) were significantly decreased (*p*-value < 0.05) when MSCs were cultured in AML-CM (i.e. MSC/AML-CM) compared with cultures of MSCs in medium alone.

| **Gene Name** | **Protein Name** |
| --- | --- |
| ADAMTS1 | A disintegrin and metalloproteinase with thrombospondin motifs 1 |
| ADAMTSL1 | ADAMTS-like protein 1 |
| ANGPTL4 | Angiopoietin-related protein 4 |
| ANTXR1 | Anthrax toxin receptor 1 |
| CLMP | CXADR-like membrane protein |
| CNN3 | Calponin-3 |
| COL10A1 | Collagen alpha-1(X) chain |
| COL16A1 | Collagen alpha-1(XVI) chain |
| COL6A2 | Collagen alpha-2(VI) chain |
| DCBLD2 | Discoidin, CUB and LCCL domain-containing protein 2 |
| DNAJC3 | DnaJ homolog subfamily C member 3 |
| ENG | Endoglin |
| EXTL2 | Exostosin-like 2 |
| FAP | Prolyl endopeptidase FAP |
| FBN2 | Fibrillin-2 |
| FLRT2 | Leucine-rich repeat transmembrane protein FLRT2 |
| FN1 | Fibronectin |
| FST | Follistatin |
| FSTL3 | Follistatin-related protein 3 |
| GALNT5 | Polypeptide N-acetylgalactosaminyltransferase 5 |
| GNG12 | Guanine nucleotide-binding protein G(I)/G(S)/G(O) subunit gamma-12 |
| HMCN1 | Hemicentin-1 |
| LOXL3 | Lysyl oxidase homolog 3 |
| LRRC15 | Leucine-rich repeat-containing protein 15 |
| MAN1B1 | Endoplasmic reticulum mannosyl-oligosaccharide 1,2-alpha-mannosidase |
| MCAM | Cell surface glycoprotein MUC18 |
| MFAP4 | Microfibril-associated glycoprotein 4 |
| NTM | Neurotrimin |
| PAM | Peptidyl-glycine alpha-amidating monooxygenase |
| PBRM1 | Protein polybromo-1 |
| PCDH9 | Protocadherin-9 |
| PDGFRB | Platelet-derived growth factor receptor beta |
| PLAT | Tissue-type plasminogen activator |
| PLOD2 | Procollagen-lysine,2-oxoglutarate 5-dioxygenase 2 |
| POSTN | Periostin |
| POSTN | Periostin |
| POSTN | Periostin |
| PRG4 | Proteoglycan 4 |
| PRKCDBP | Protein kinase C delta-binding protein |
| PRSS23 | Serine protease 23 |
| S100A13 | Protein S100-A13 |
| SDC1 | Syndecan-1 |
| THY1 | Thy-1 membrane glycoprotein |
| TSPAN4 | Tetraspanin-4 |
| ULBP2 | NKG2D ligand 2 |

**Table S8.** The 15-ECM signature identified in primary AML cells by gene expression analyses and having a prognostic impact in human AML; a comparison with the results from the present proteomic studies where 10-ECM signature members and CD44 were quantified. .

|  |  |  | **Number of AML Cultures with Detectable Levels** | | **MSC/AML-CM Versus AML-CM** | |
| --- | --- | --- | --- | --- | --- | --- |
| **Protein Name** | **Gene Name** | **MSC10 Release ^§^** | **AML** | **MSC/AML-CM** | **Median FC (log_2_) ^#^** | **Paired *t*-test** |
| Disintegrin and metalloproteinase domain-containing protein 17 | ADAM17 | - | 6 | 14 | 1.26 | 0.0070 |
| Chitinase-3-like protein 1 | CHI3L1 | + | 8 | 12 | 0.31 | Ns |
| Collagen alpha-1(XVIII) chain;Endostatin | COL18A1 | ++ | 5 | 40 | 1.91 | Ns |
| Neutrophil defensin 3 | DEFA1/3 | - | 22 | 22 | -0.28 | Ns |
| Neutrophil elastase | ELANE | - | 30 | 32 | -0.46 | 0.039 |
| EMILIN-2 | EMILIN2 | + | 21 | 16 | -0.84 | 0.00022 |
| Galectin-3 | LGALS3 | + | 26 | 21 | 0.45 | Ns |
| Neutrophil collagenase | MMP8 | - | 10 | 8 | 0.13 | Ns |
| Matrix metalloproteinase-9 | MMP9 | + | 29 | 21 | 0.57 | Ns |
| Myeloblastin | PRTN3 | - | 31 | 33 | 0.13 | Ns |
| * CD44 antigen | CD44 | ++ | 39 | 35 | 0.09 | Ns |

^§^ The MSC was characterized as undetectable (-), low (+, detected only for some of the MSC cultures) and high (++; detected in all seven MSC cultures). **^#^** Positive values indicate increased levels in cultures prepared with MSC/AML-CM. As criterion for significant protein release, a 2-fold increase or 4-fold decrease was required in addition to *p*-value < 0.05. * CD44 is not a member of the 15-ECM signature, but is known to bind many ECM proteins.


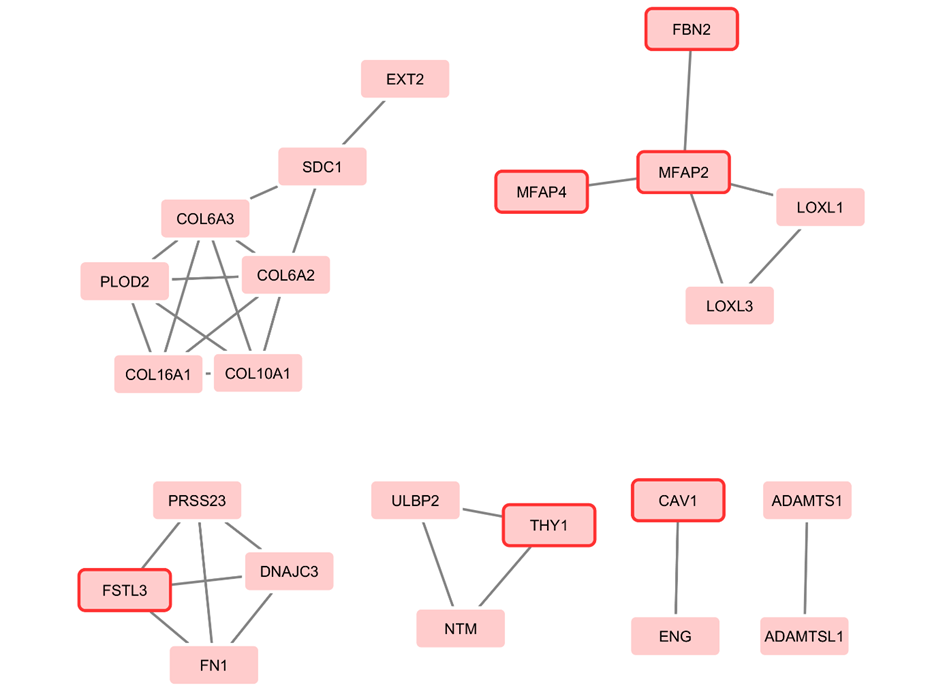


**Figure S1.** Protein interaction networks based on the 61 constitutively MSC-released proteins that were quantified in ≥4 MSCs and only by ≤2 primary AML cells derived from the 40 leukemia patients. Among the 61 proteins, 23 were connected in 5 networks. Protein nodes marked with red borders were only quantified in the MSC supernatants. The interaction network was generated by the String database and imported to Cytoscape for color coding. Functional enrichment analysis (in String) of proteins in the network showed annotated Uniprot keywords as Secreted, Signal and Extracellular Matrix.

**
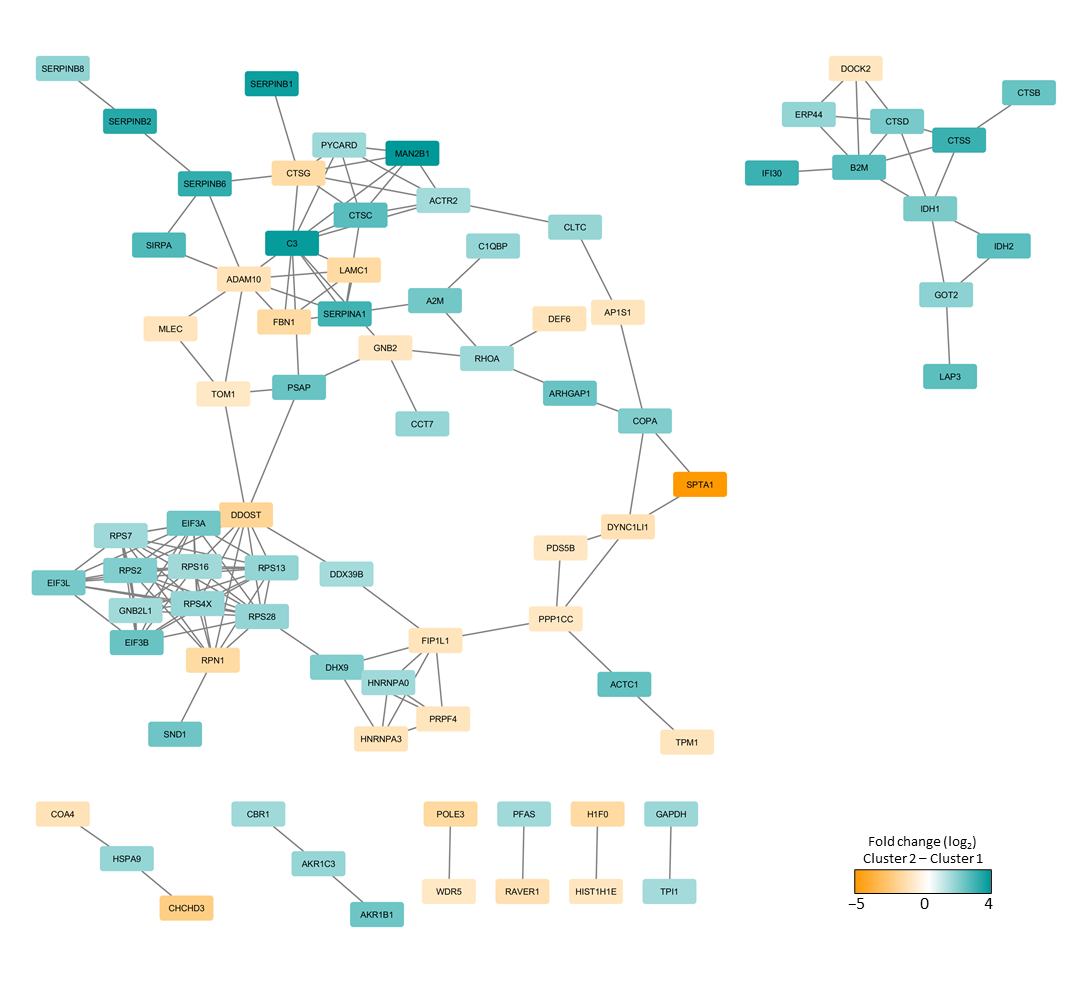
**

**Figure S2.** Protein interaction networks based on 144 proteins with significantly different abundance when comparing the AML-CM samples forming cluster 1 and 2 in Figure 4. The interaction network was generated by the String database and imported into Cytoscape for color coding. Turquoise color indicates higher abundance in cluster 2 (brown protein cluster), and orange color indicates higher abundance in cluster 1 (yellow protein cluster).


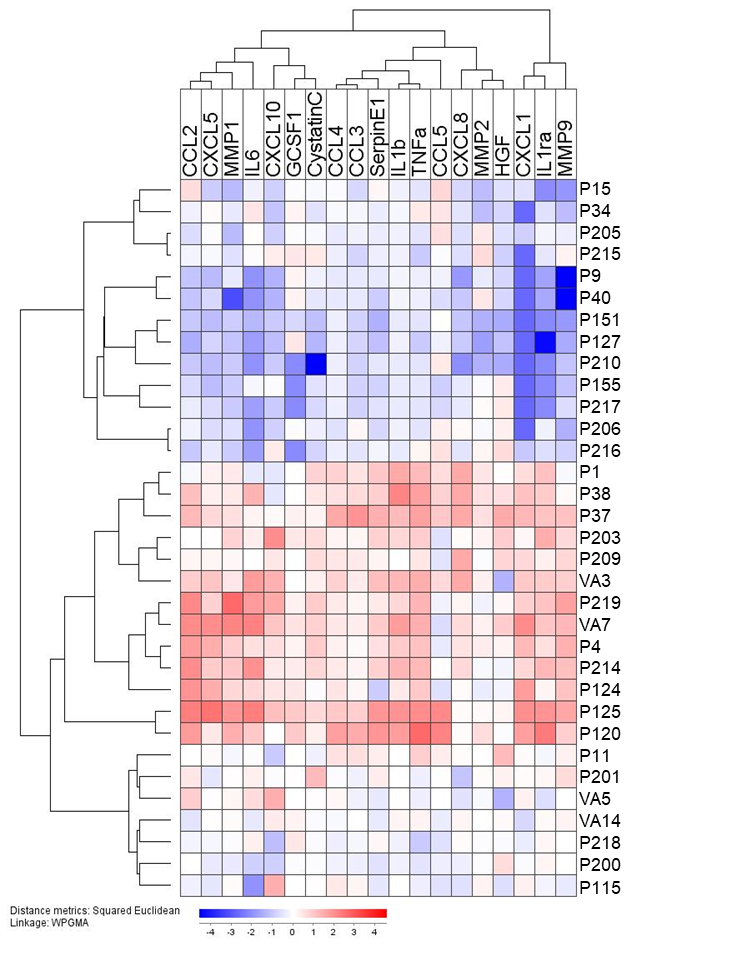


**Figure S3.** Unsupervised hierarchical clustering of 17 cytokines and 2 protease inhibitors (serpinE1 and cystatinC). The cluster includes 33 of the 40 patients, for which all the mediator concentrations were measured. Supernatants were collected from AML cells cultured for 48h in Stem Span SFEM™ medium (Stem Cell Technologies; Vancouver, BC, Canada) in 24-well culture plates (1 × 10^6^ cells per mL; Nunclon, Roskilde, Denmark), and were stored at −80 °C prior to analysis. Protein levels were determined by Luminex analyses and enzyme-linked immunosorbent assays (ELISA) (R&D Systems; Minnesota, MN). The concentrations were median normalized and log(10) transformed prior to the hierarchical clustering analysis (Euclidean clustering in J-Express 2012 software, MolMine AS; Bergen, Norway). The patients could be divided into three clusters/subsets: low- (upper cluster), high-release- (middle), and intermediate release levels (lower).

**
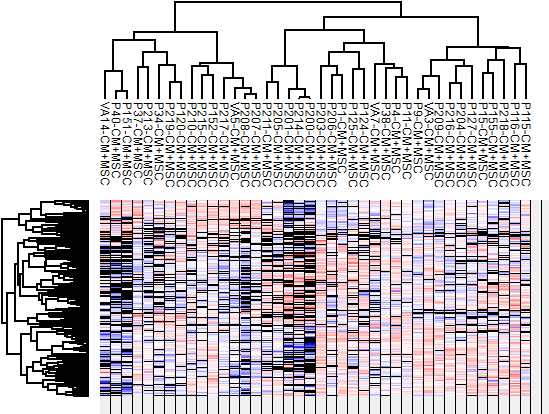
**

**Figure S4.** Identification of AML patient subsets based on the protein intensity (log_2_-transformed and Z-scored) in supernatants derived from MSC cultures supplemented with AML-CM; an unsupervised hierarchical cluster analysis including all 40 patients. The cells were cultured for 48 hours in serum-free medium before supernatants were harvested and the proteomic analyses performed. The analysis was based on those protein that could be detected for at least 50% of the patients, and the protein values were Z-scored. The patients clustered into three main clusters as can be seen from the upper part of the figure. These three patient clusters are referred to as left middle and right cluster in Figure 5 in the article.

**
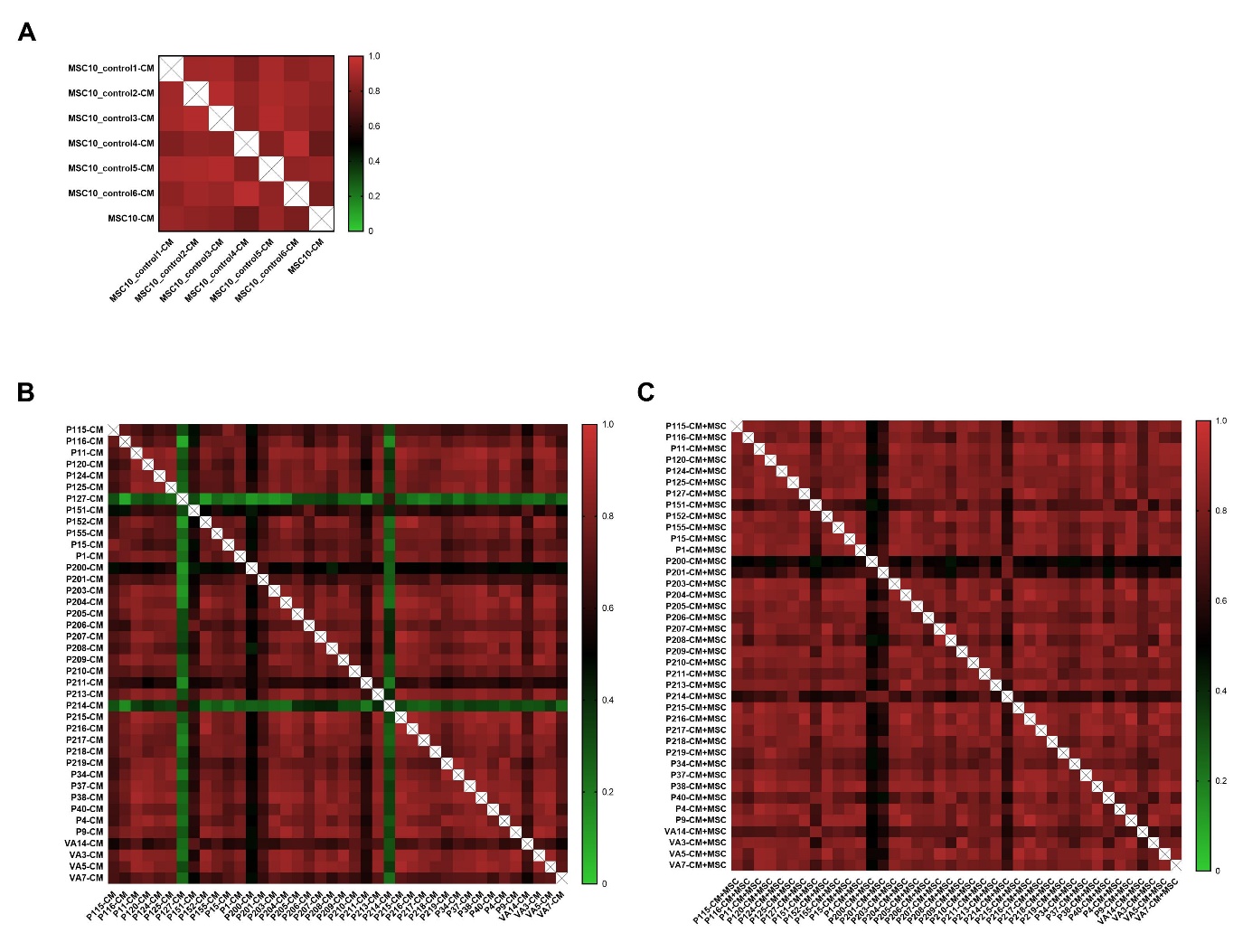
**

**Figure S5.** A correlation plot comparing the protein expression in seven independent cultures of our MSCs derived from a healthy donor. The figure presents a Pearson correlation plot of the quantified proteins. The analysis shows that there was a considerable overlap between the protein expression in the various cultures, i.e. the protein release profile of these MSCs were highly reproducible. MSC10-CM was cultured in a T25 flask, like the AML-CM samples, while MSC10_control-CM (1-6) were cultured in wells like the MSC/AML-CM samples.
